# Supplementary material for: Mutations in FAM50A suggest that Armfield XLID syndrome is a spliceosomopathy
Source: Nat Commun. 2020 Jul 23;11:3698. doi: 10.1038/s41467-020-17452-6 (PMC7378245; doi:10.1038/s41467-020-17452-6)
Supplement: Supplementary file 1 — Supplementary Information [file 41467_2020_17452_MOESM1_ESM.pdf]

Supplementary Information

Mutations in *FAM50A* suggest that Armfield XLID syndrome is a spliceosomopathy

Lee et al.

Supplementary Figures

Supplementary Figure 1

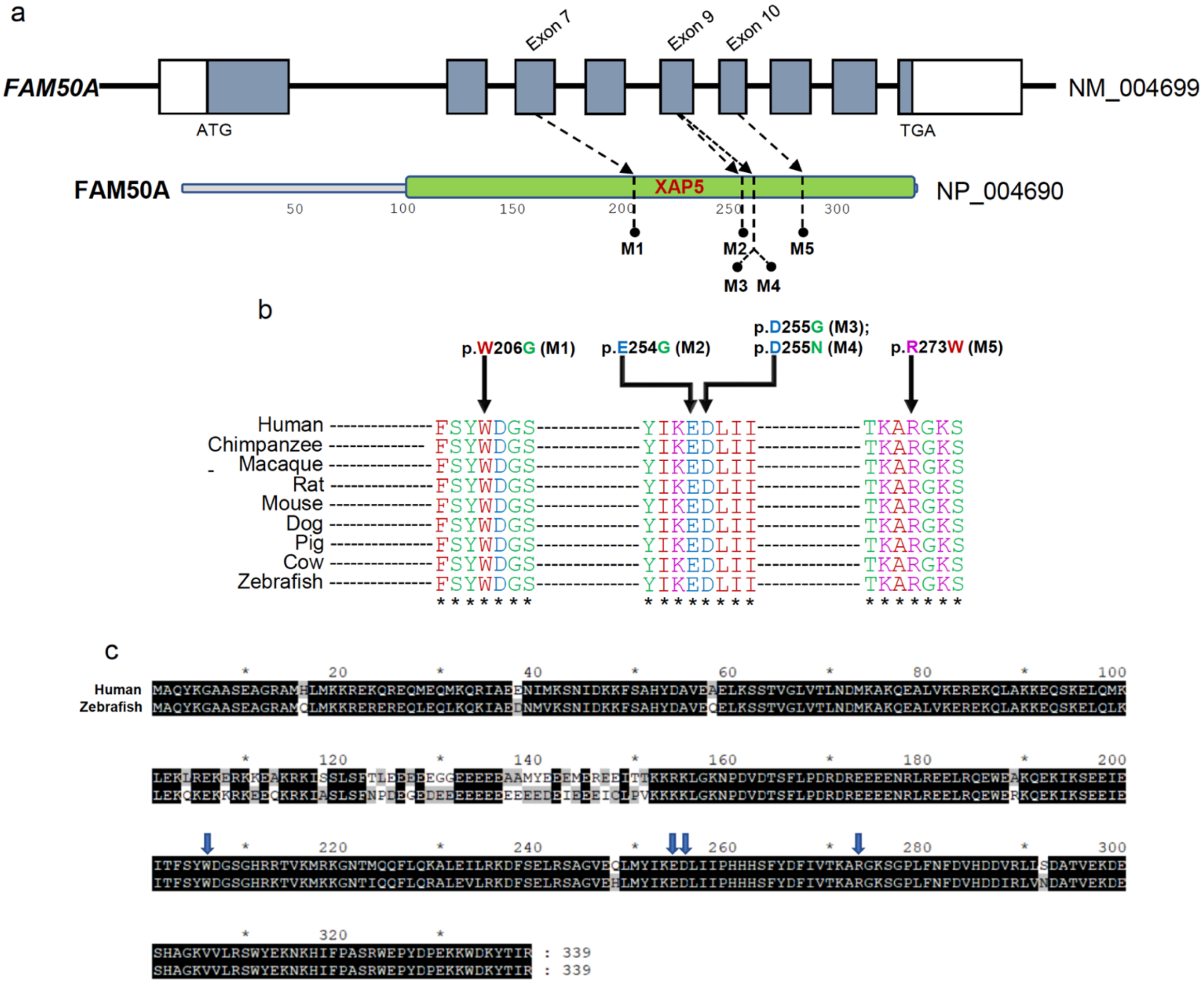

Supplementary Figure 1. *FAM50A* missense variants are conserved across eukaryotic phyla.

a) Top, schematic of the *FAM50A* gene on Xq28, showing exons (boxes) and introns (black horizontal line); coding regions, blue; untranslated regions, white. Bottom, schematic of the FAM50A protein, and location of the five mutations on the XAP5 domain.

b) Multiple sequence alignment generated with Clustal Omega shows that all five *FAM50A* mutations are located in three regions conserved among vertebrates. Amino acid color scheme: Red- hydrophobic or aromatic (AVFPMILWY); blue- acidic (DE); magenta- basic (RHK); green- hydroxyl, amine, basic and glutamine (STYHCNGQ). Consensus symbols, an asterisk (*) indicates positions which have a single, fully conserved residue.

c) Alignment of human FAM50A (NP_004690.1) and zebrafish Fam50a (NP_001017636.1) protein sequences generated with ClustalX. The zebrafish ortholog encodes a 339-amino-acid protein, with a high level of similarity (94%) to the human FAM50A protein. Black, conserved residue; blue arrows, location of variants identified in affected males.

Supplementary Figure 2

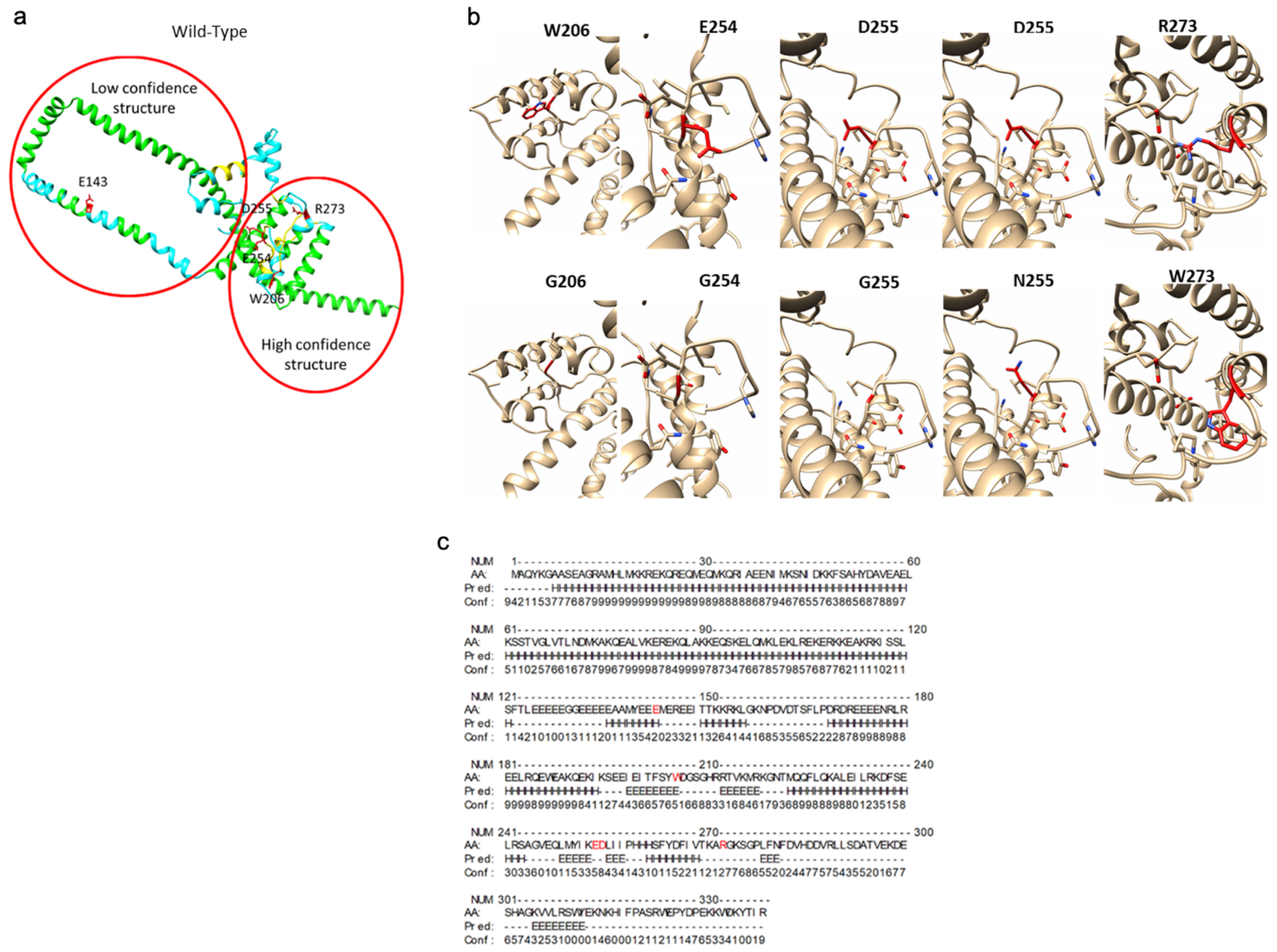

Supplementary Figure 2. *FAM50A* variants are predicted to disrupt protein secondary structure.

a) Predicted models for FAM50A protein generated with I-TASSER, and validated with secondary structure element (SSE) predictions generated with YASPIN (Wild Type). The prediction of the secondary structure is mapped on the model: helix, green; beta strand, yellow; and coil, cyan. The prediction indicates that residues from 1 to 200 are mostly helix, highly consistent with the 3D model except some small fragments. As the model of FAM50 is mostly composed of the coils and helix, the major inconsistency arises from the predicted beta strand regions. Overall, only a small fragment of the structure was predicted to be beta strand (around 30 residues), thus not significantly affecting the quality of the model.

b) Close ups of the predicted models showing the effects of each mutation on the protein structure of FAM50A. The mutation sites are colored as red with side chain (Wild Type and Mutant).

c) Prediction of secondary structure for the FAM50A protein generated with YASPIN. Residues with mutations are colored as red. Row labels indicate the residue number, amino acid sequence, predicted secondary structure (H, helix; E, strand; -, coil) and confidence score (0=low, 9=high).

Supplementary Figure 3

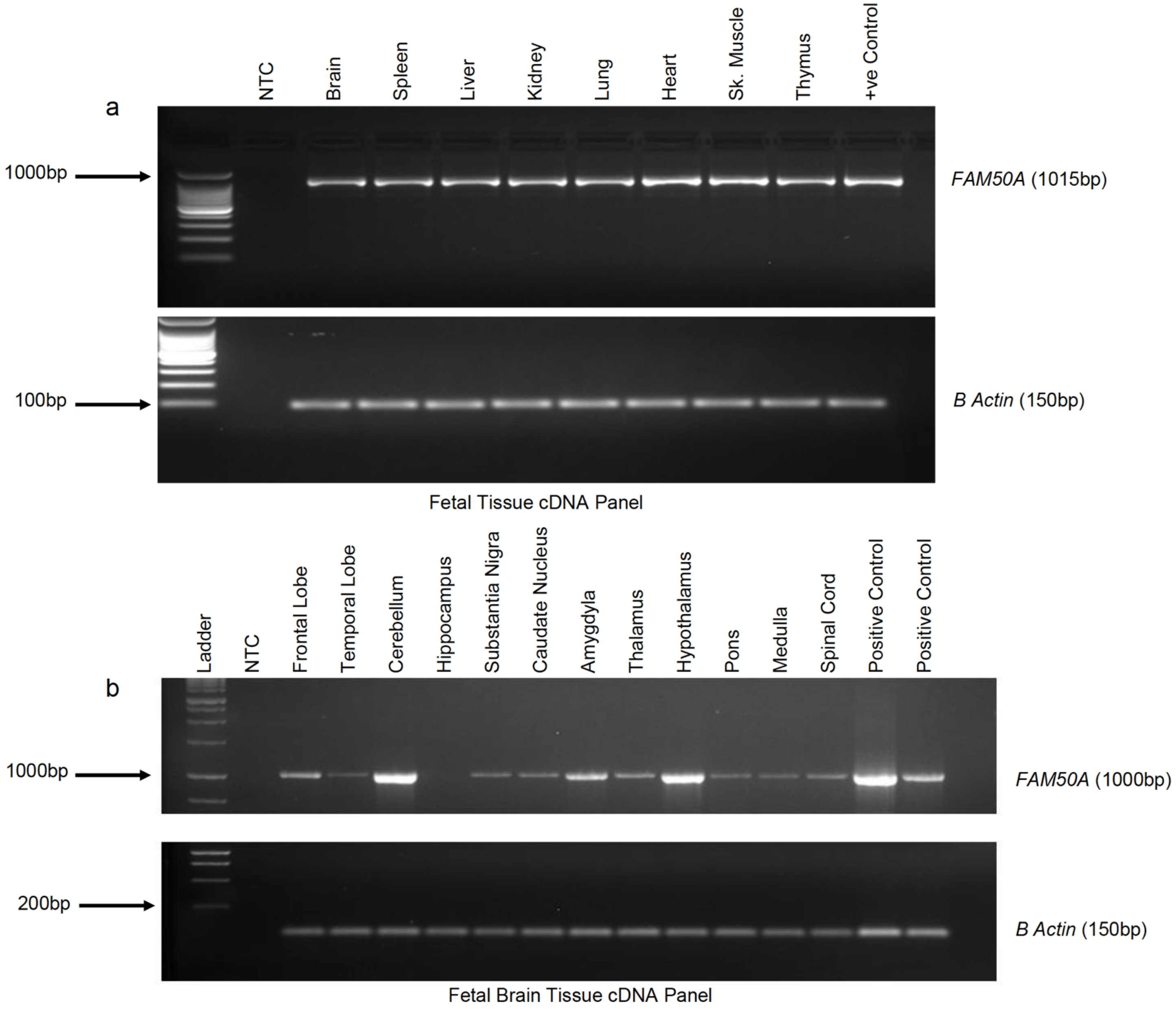

Supplementary Figure 3. *FAM50A* expression is ubiquitous.

a) Agarose gel images showing migration of RT-PCR products from multiple fetal tissues. *FAM50A* = 1015 bp. β-*Actin* = 150 bp. NTC, no-template control.

b) Agarose gel images showing migration of RT-PCR products from the Rapid Scan Human Fetal Brain panel. *FAM50A* = 1015bp, β-Actin = 150 bp.

Both experiments were repeated twice, with similar results; Uncropped gel images are shown in Source Data.

Supplementary Figure 4

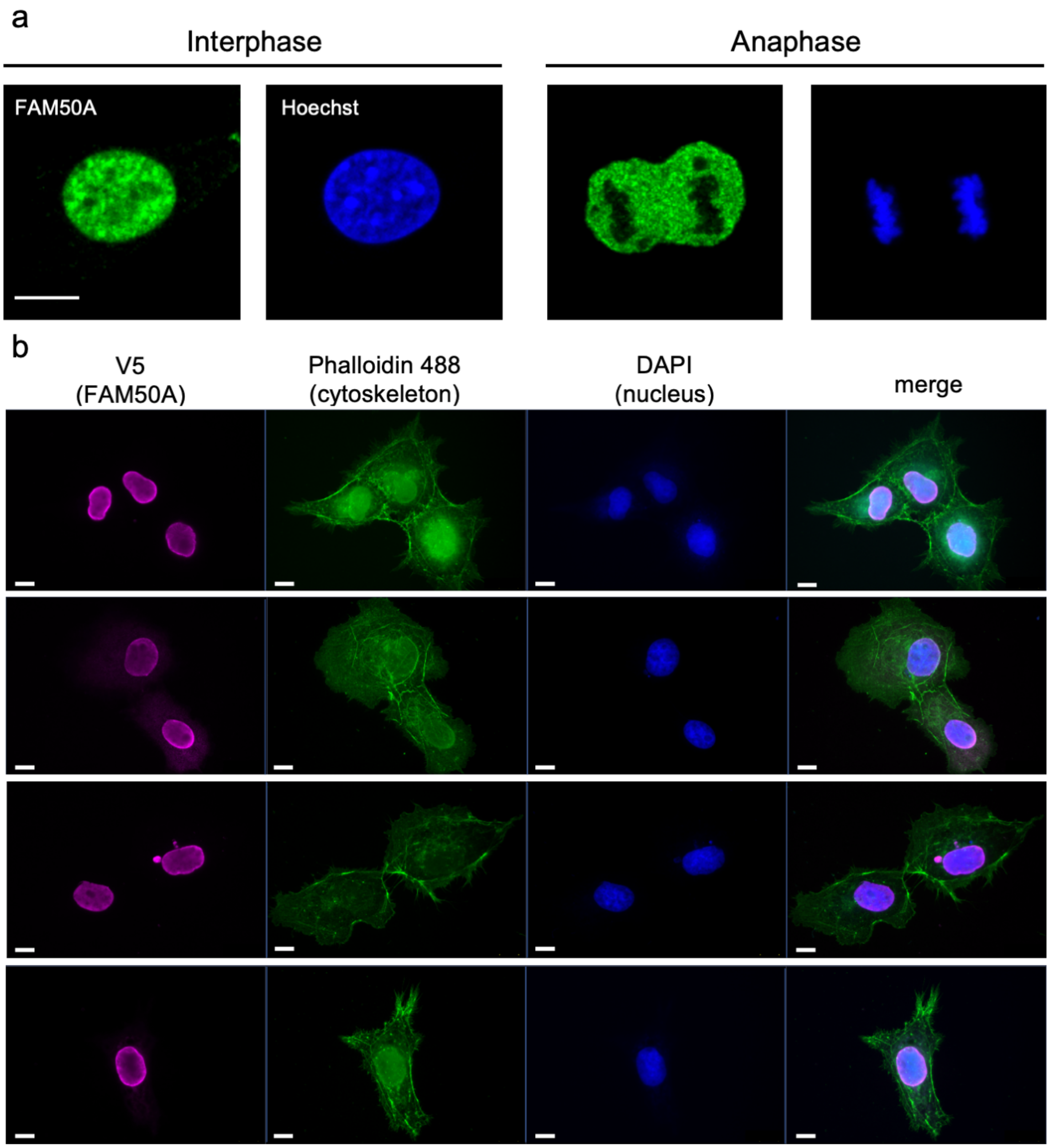

Supplementary Figure 4. FAM50A protein localizes to the nucleus in mammalian cells.

a) NIH/3T3 cells were immunostained with anti-FAM50A antibody to detect endogenous protein. Representative cells are shown. n=31 cells examined, each with similar results. Scale bar: 10 μm, with equivalent sizing across panels.

b) COS-7 cells were transiently transfected with a C-terminally V5 tagged FAM50A plasmid (either WT or *FAM50A* mutations p.Trp206Gly, p.Glu254Gly, p.Asp255Gly). Cells were fixed and immunostained with mouse anti-V5 monoclonal antibody and fluorescence signal was detected at 60X magnification. Experiments were repeated three times, with similar results. Scale bar: 10 μm, with equivalent sizing across panels.

Supplementary Figure 5

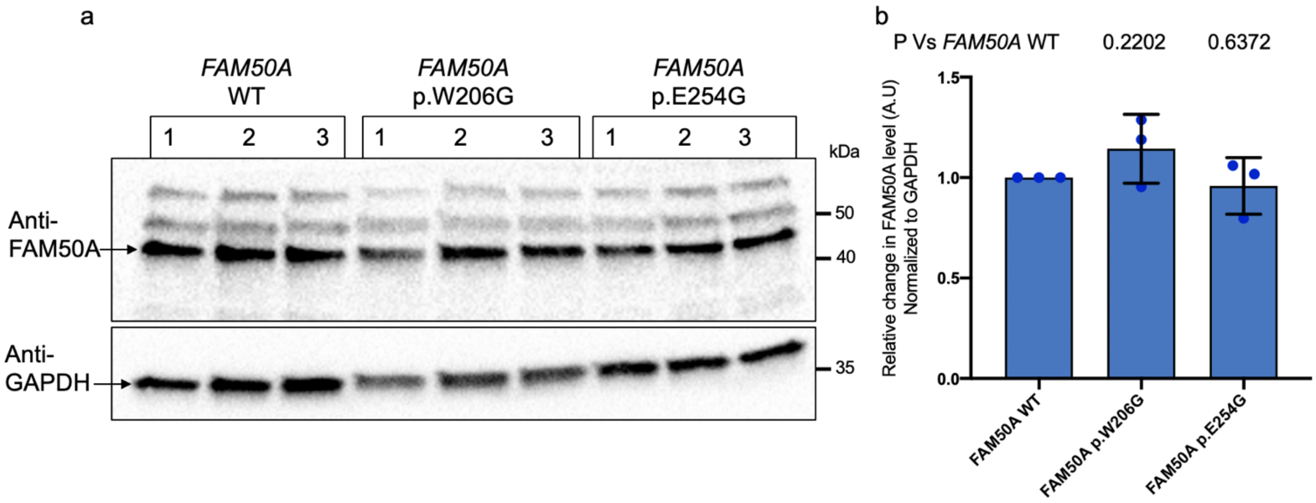

Supplementary Figure 5. FAM50A patients showed no detectable differences in FAM50A protein level compared to control.

a) Western blot was performed on 23 µg whole-cell protein lysate isolated from lymphocyte cell lines derived from individuals with *FAM50A* mutations (Family K9648, p.Trp206Gly; Family K9656, p.Gly254Gly) and an unrelated male control of European origin (FAM50A WT). The immunoblot was performed in three independent biological replicates for each condition using rabbit anti-FAM50A antibody (Novus Biologicals; Cat # NBP1-89344; 1: 900 dilution) and mouse anti-GAPDH antibody (Santa Cruz Biotechnology, Cat# sc-47724; 1:3000 dilution). FAM50A and GAPDH are indicated with black arrows. Uncropped blot image is shown in Source Data.

b) Chemiluminescence signal was quantified using Image Studio Lite and normalized to GAPDH in each condition. FAM50A protein abundance is shown relative to male control and statistical differences were calculated using an unpaired student's t-test (two-sided). Data are presented as mean values +/- standard deviation; (n= 3 biological replicates per condition).

Supplementary Figure 6

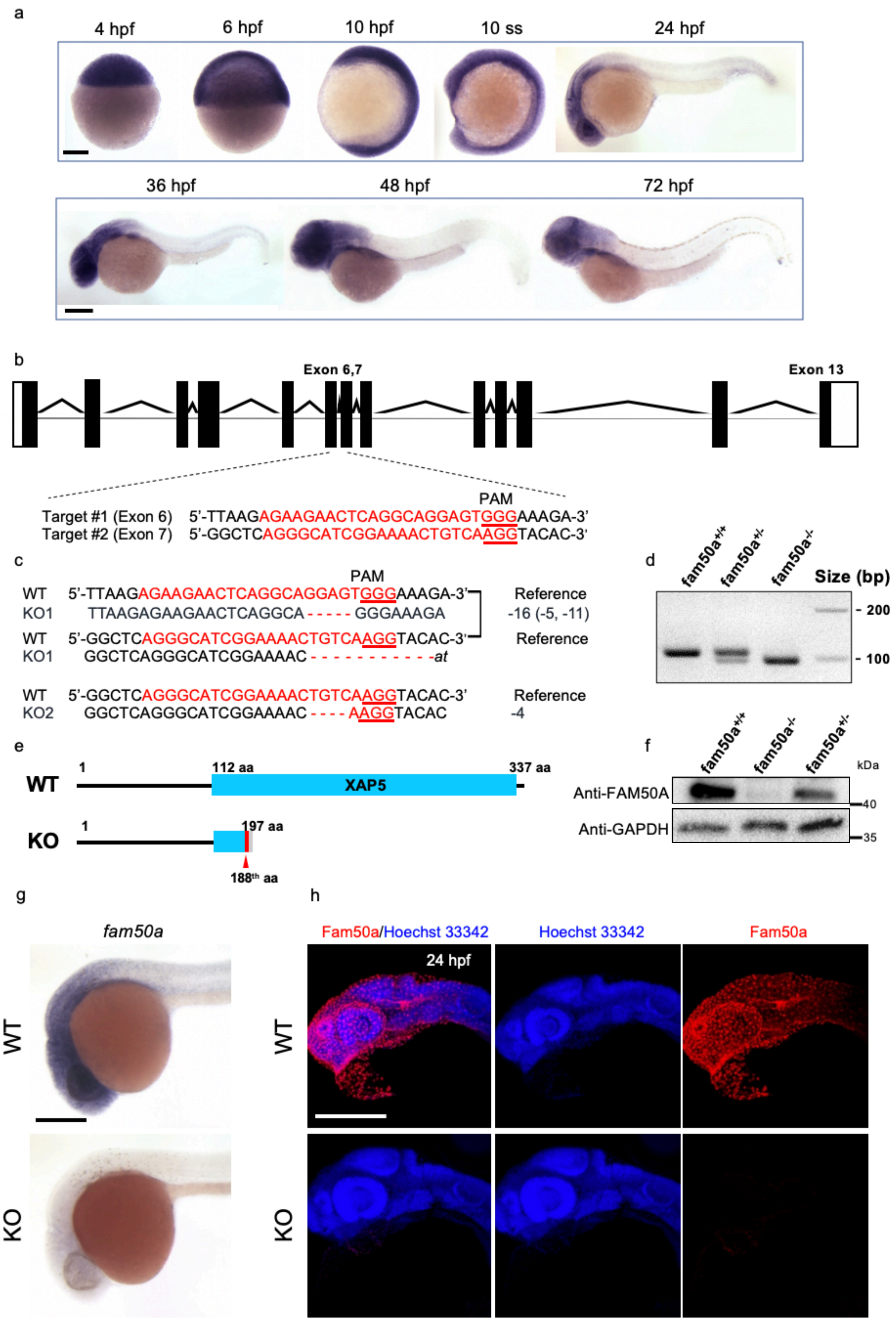

Supplementary Figure 6. Generation and validation of the *fam50a* KO zebrafish model.

a) Representative images of wholemount RNA *in situ* (WISH) to monitor expression of *fam50a* during zebrafish development from 4 hours post-fertilization (hpf) through 72 hpf. n=5 embryos examined at each stage; each experiment was repeated three times with similar results. Scale bar: 200 μm, with equivalent sizing across panels.

b) Schematic showing the genomic structure of zebrafish *fam50a* (GRCz10: GENSDART00000037501.8) and target sites for knock out (KO) generation by CRISPR-Cas9. Black boxes, coding regions; white boxes, untranslated regions; black lines, introns.

c) Targeted sequence for CRISPR guide RNAs. Identification of 4 bp and compound 11+5 bp deletion (KO) mutants. Phenotyping data in this study correspond to KO1.

d) Representative agarose gel image of PCR products used to genotype *fam50a* zebrafish mutants. n=20 embryos; repeated 12 times with similar results; uncropped images are shown in the Source Data.

e) Predicted structure of WT and *fam50a* KO mutant proteins. The compound 11+5 bp deletion resulted in a frame-shifting mutation with premature termination in the XAP5 domain.

f) FAM50A western blot was performed on protein lysates isolated from zebrafish heads. Zebrafish heads were cross-matched with genotyping performed on DNA extracted from zebrafish tails and 20 embryos were pooled per genotype to isolate total protein lysate. A total of 8 μg/lane protein was migrated on 4-15% polyacrylamide gels and anti-FAM50A rabbit poly-clonal antibody (Novus Biologicals; Cat # NBP1-89344; 1:900 dilution) was used to detect native protein. GAPDH was used as loading control. Repeated twice, with similar results; uncropped images are shown in the Source Data.

g) Representative images of WISH demonstrate loss of *fam50a* expression in KO zebrafish at 24 hpf. n=19 embryos assessed; repeated twice with similar results. Scale bar: 200 μm, equivalent sizing across panels.

h) Wholemount immunofluorescence of Fam50a protein in WT and *fam50a* KO mutant zebrafish at 24 hpf. Confocal image shows Fam50a (red), and nuclei counterstained with Hoechst 33342 (blue). Fam50a protein was not detectable in *fam50a* KO embryos. n=12 embryos assessed with similar results. Scale bar: 200 μm, equivalent sizing across panels.

Supplementary Figure 7

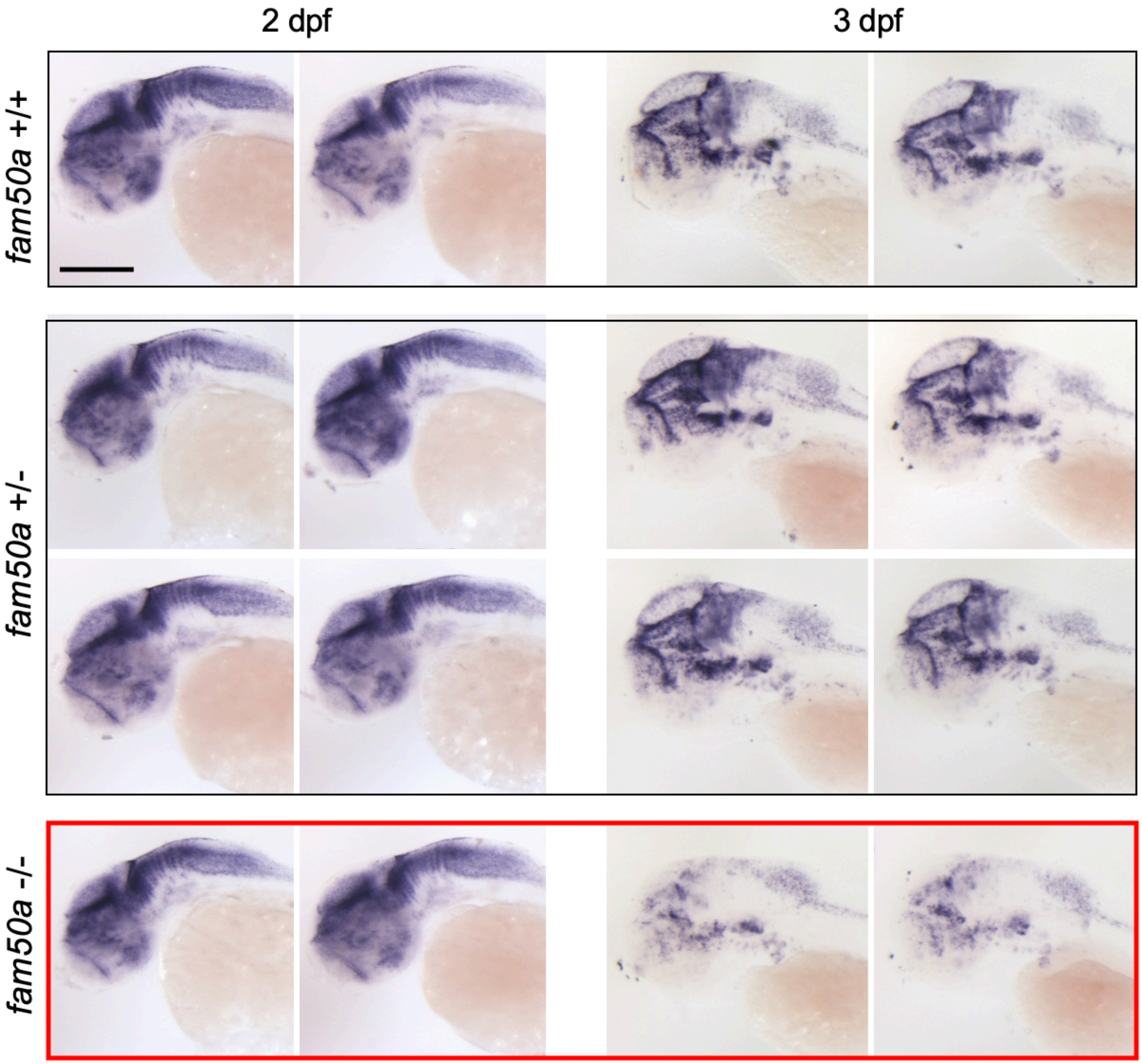

Supplementary Figure 7. Validation of zebrafish RNAseq data using RNA *in-situ* hybridization assays. *In situ* expression of *her4.1* in *fam50a* KO zebrafish larvae ($^{-/-}$) is similar to wild-type (WT, $^{+/+}$) and *fam50*$^{+/-}$ (heterozygotes) at 2 days post-fertilization (dpf) but depleted at 3 days post-fertilization. Images of representative larvae are shown; related to Figure 2c. Number of embryos evaluated: 2 dpf, n=24; 3 dpf, n=21; repeated twice with similar results. Scale bar; 200 μm, with equivalent sizing across panels.

Supplementary Figure 8

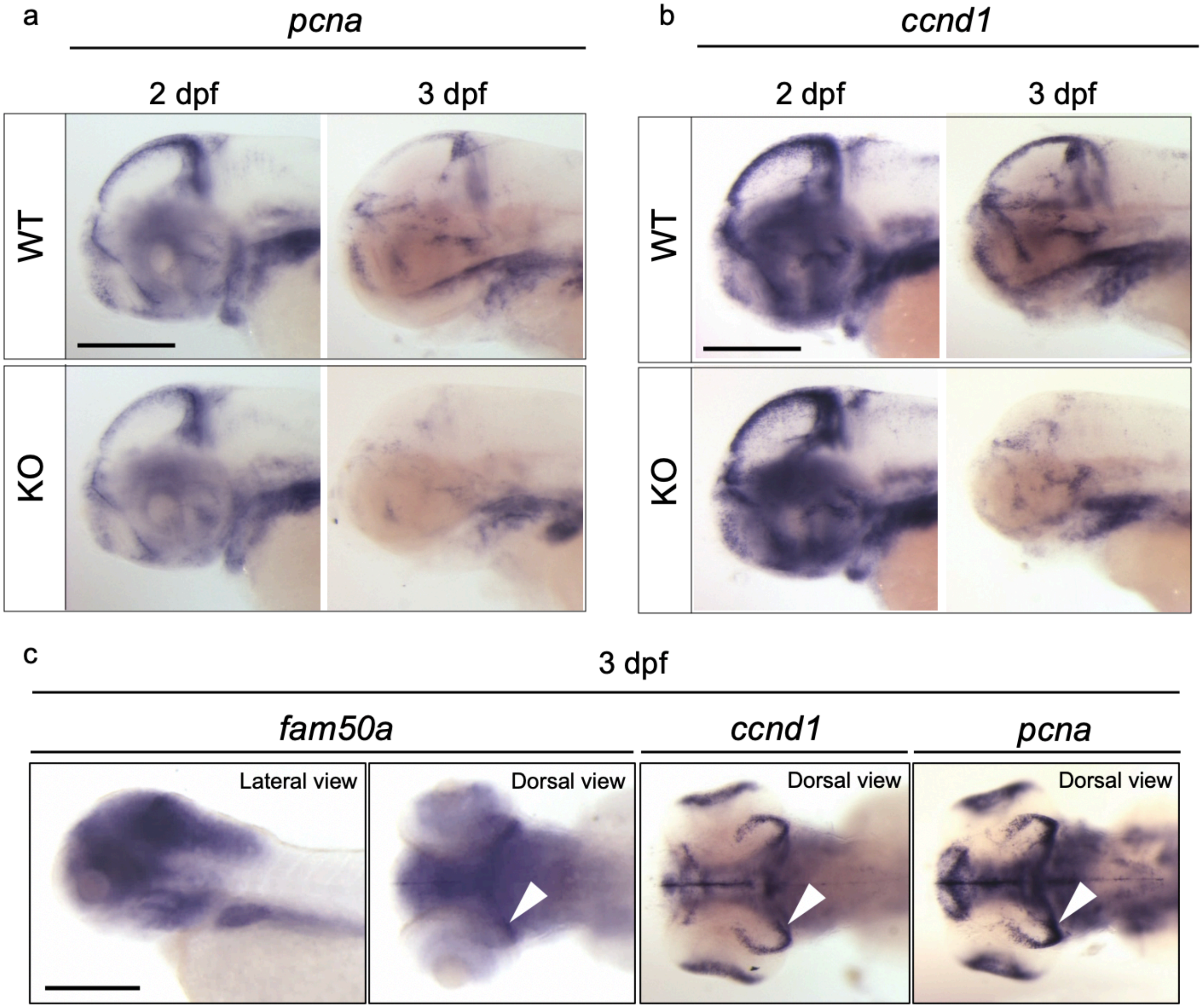

Supplementary Figure 8. *In situ* expression of cell proliferation markers in *fam50a* KO zebrafish larvae is depleted.

Cell proliferation markers showed no detectable change in *fam50a* KO zebrafish at 2 days post-fertilization (dpf), however, significant reduction was noticed at 3 dpf.

a) Proliferating cell nuclear antigen (*pcna*); 2 dpf, n=16; 3 dpf, n=18 and

b) Cyclin D1 (*ccnd1*) mRNA expression. 2 dpf, n=19; 3 dpf, n=16.

c) Comparison of *fam50a, ccnd1* and *pcna* expression in WT larvae at 3 dpf show a similar pattern of concentrated expression in the proliferative zone of the midbrain (white arrow). *fam50a*, n=5; *ccnd1*, n=16; *pcna*, n=18. Experiments were repeated two times, with similar results. Scale bars; 200 μm, with equivalent sizing across panels.

Supplementary Figure 9

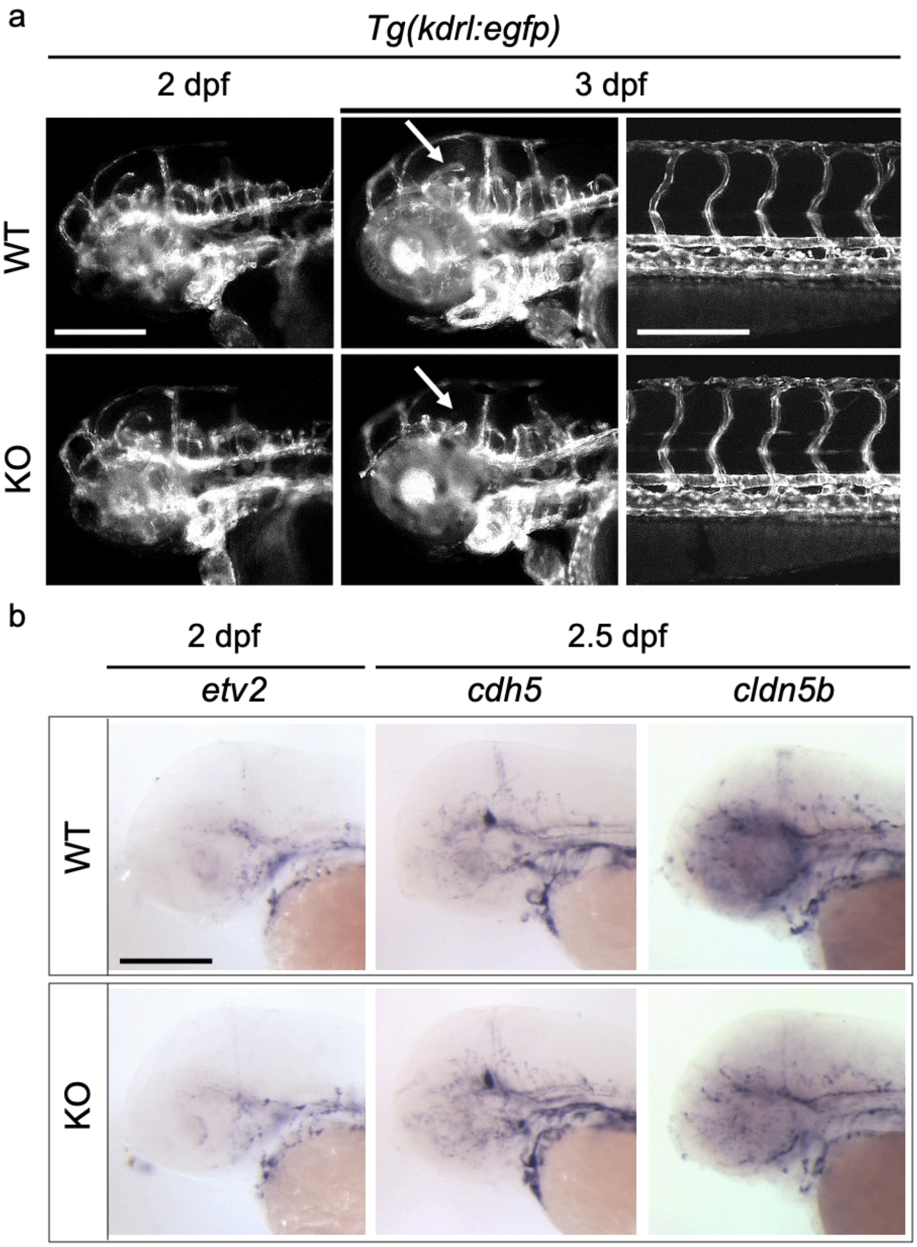

Supplementary Figure 9. *fam50a* KO zebrafish larvae display normal blood vessel patterning.

a) Endothelial-specific expression of GFP in the transgenic *tg(kdrl:egfp);fam50a* KO zebrafish at 2 days post-fertilization (dpf) and 3 dpf. Blood vessel defects are first detectible in the midbrain region of 3 dpf KO zebrafish larvae (arrows). However, intersegmental vessels in the trunk region are relatively normal in the 3 dpf KO zebrafish, compared to WT. Number of larvae assessed, with similar results: 2 dpf, n=24; 3 dpf, n=11. Scale bar: 200 μm, with equivalent sizing across panels.

b) Wholemount *in situ* analysis of vascular markers in *fam50a* KO zebrafish larvae shows relatively normal mRNA expression at 2- and 2.5-day post-fertilization (dpf). Molecular markers used: *etv2*, ETS variant 2; *cdh5*, Cadherin 5; and *cldn5b*, Claudin 5b. Number of larvae assessed, with similar results: *etv2*, n=17; *cdh5*, n=18; *cldn5b*, n=28. Blood vessels were assessed in transgenic zebrafish in two independent replicate experiments and validated once by *in situ* expression of vascular markers. Scale bar: 200 μm, with equivalent sizing across panels.

Supplementary Figure 10

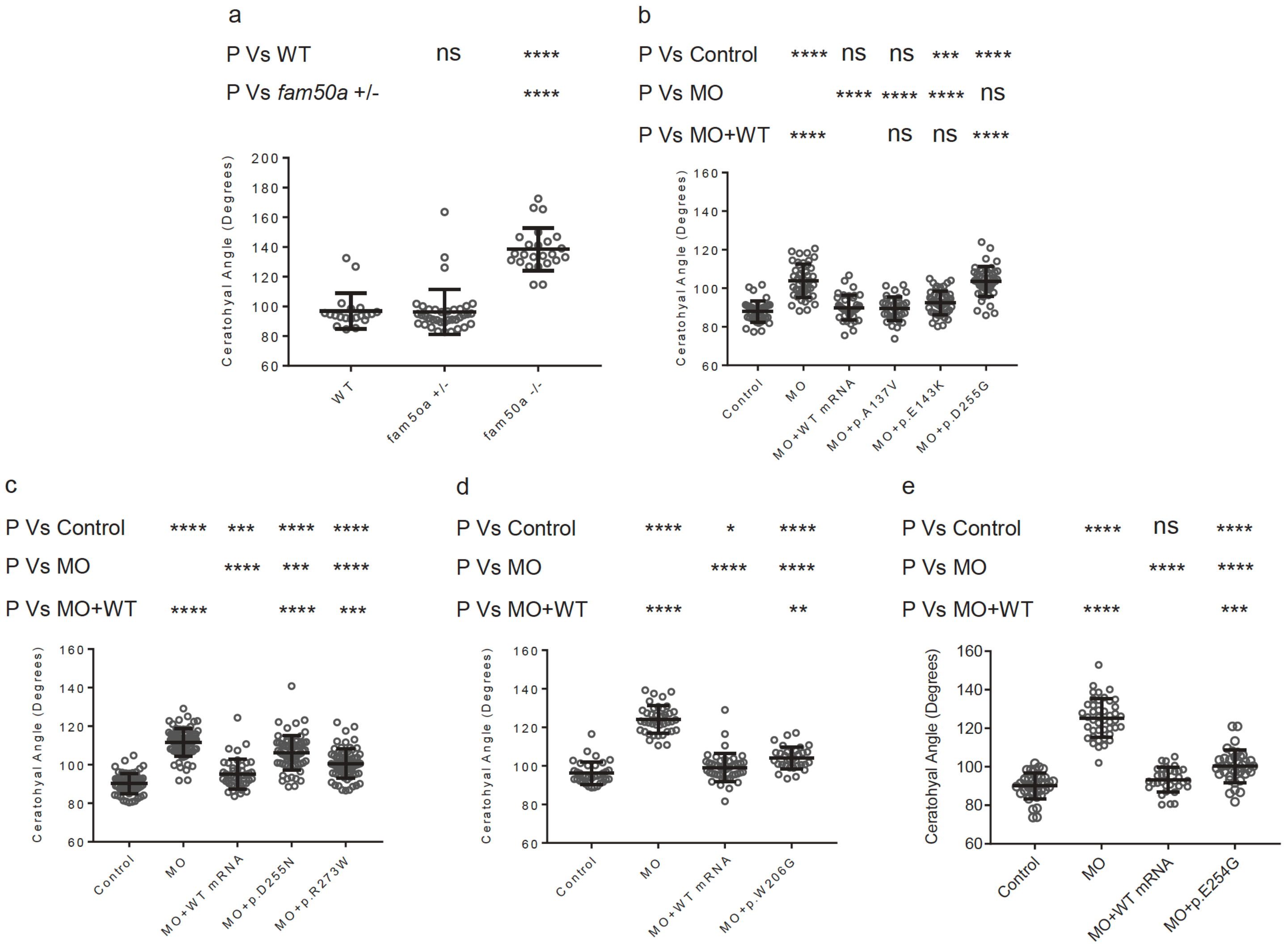

Supplementary Figure 10. Dot plots representing ceratohyal angle measurements.

Dot plots correspond to individual *fam50a* KO and *in vivo* complementation phenotyping studies of craniofacial patterning (as measured by ceratohyal angle). Statistical differences were calculated using an unpaired student's t-test (two-sided); exact p-values and number of larvae are listed in Supplementary Table 4. *, **, ***, **** indicate p<0.05; 0.01; 0.001; and 0.0001, respectively. ns, not significant. Data are presented as mean values +/- standard deviation, experiments were repeated two times, with similar results (a) corresponds to Figure 3b; and (b, c, d and e) correspond to Figure 3c.

Supplementary Figure 11

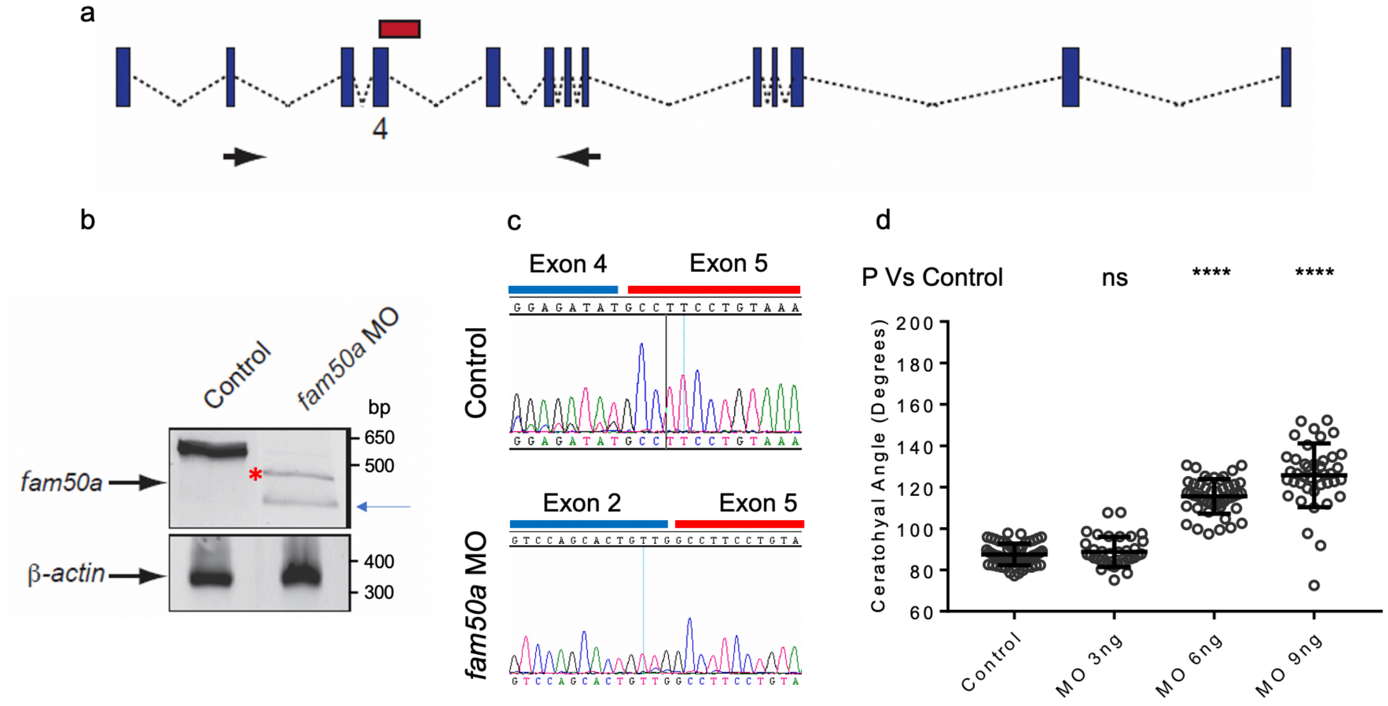

Supplementary Figure 11. *fam50a* is targeted specifically by a splice-blocking morpholino.

a) Schematic of the *D. rerio fam50a* locus showing exons as blue boxes and introns as black dotted lines. The morpholino (MO) sequence is shown as a red rectangle targeting the splice donor site of exon 4; PCR primers are indicated as black arrows flanking the MO target region.

b) Agarose gel image (2%) showing migration of *fam50a* RT-PCR products amplified from the region flanking the MO target site; red star represents deletion of exon 3 and part of exon 4, blue arrow represents deletion of exons 3 and exon 4 (see c). *β-actin* was used to control for RNA integrity. See Source Data for uncropped gel image.

c) Sequence chromatograms of amplified region flanking the MO target site in morphants indicates deletion of exon 3 and exon 4 (arrow, see panel b).

d) Injection of increasing concentrations of *fam50a* MO resulted in a dose dependent craniofacial phenotype (increase in ceratohyal angle) in zebrafish larvae at 3 dpf. Statistical differences were calculated using an unpaired student's t-test (two-sided); Exact p-values and number of larvae are listed in the Supplementary Table 4; **** indicates p<0.0001, ns, not significant; experiment repeated two times with similar results. Data are presented as mean values +/- standard deviation.

Supplementary Figure 12

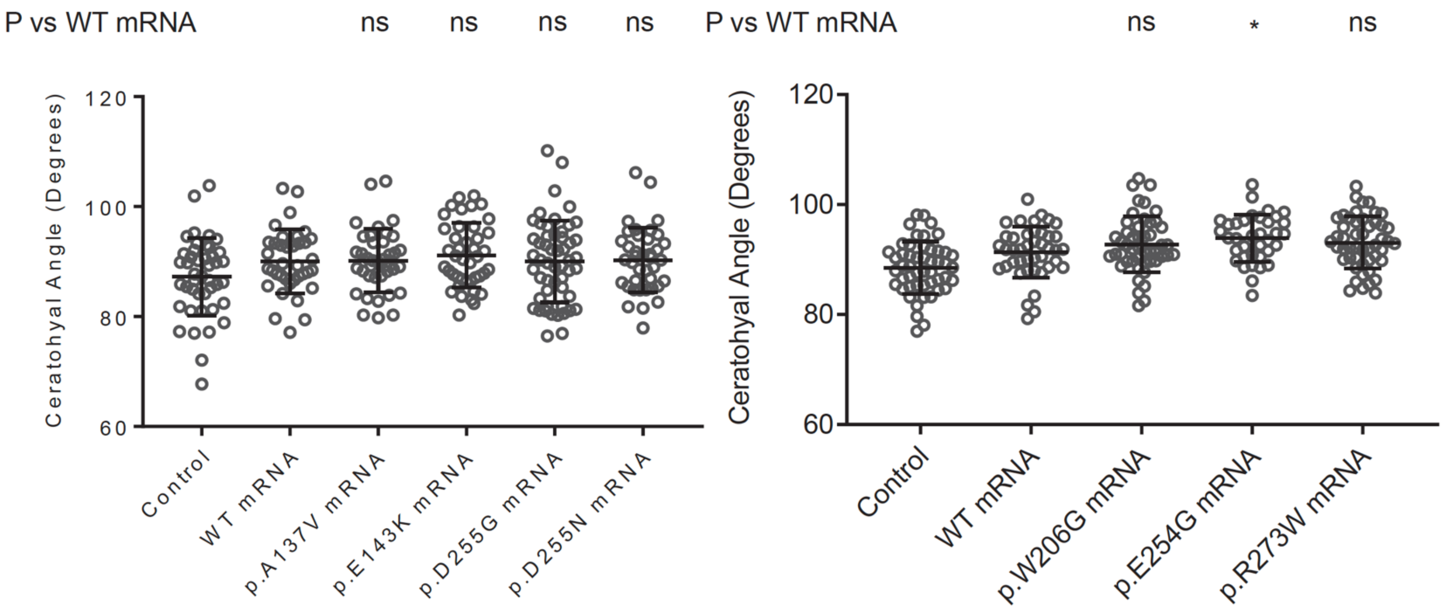

Supplementary Figure 12. Injection of human *FAM50A* mRNA does not induce a significant effect on cartilage patterning in zebrafish larvae.

Capped *FAM50A* mRNA was injected into *-1.4col1a1:egfp* zebrafish embryos at the 1-4 cell stage; larvae were imaged live at 3 days post-fertilization (dpf) using the VAST BioImager; and ceratohyal angle was measured. Statistical differences were calculated using an unpaired student's t-test (two-sided); * indicates $p<0.05$; exact p-values and number of larvae are listed in Supplementary Table 4; ns, not significant; experiment was repeated two times with similar results. Data are presented as mean values +/- standard deviation.

Supplementary Figure 13

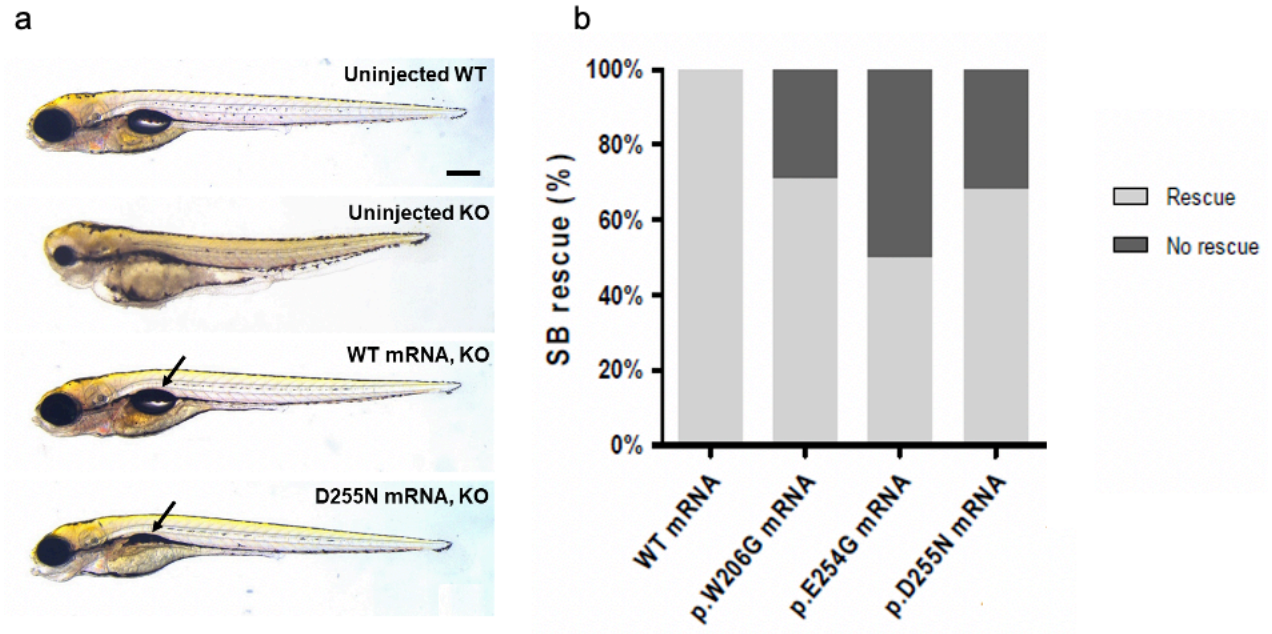

Supplementary Figure 13. Swim bladder assay in *fam50a* KO zebrafish validates pathogenicity of *FAM50A* variants.

a) Rescue experiment of *fam50a* KO zebrafish with 300 pg human WT *FAM50A* mRNA injection or patient variant mRNA injection. KO phenotypes were rescued by WT *FAM50A* expression, but only partly rescued by patient variants. Swim bladder (SB) formation at 5 dpf was used as criteria for quantification of rescue measurement (arrows). Scale bar; 200 μm, with equivalent sizing across panels.

b) Quantification of injected KO zebrafish rescued by SB formation (SB rescue, %). WT, n=24; p.Trp206Gly, n=65; p.Glu254Gly, n=46 and; p.Asp255Asn, n=88, repeated with similar results.

Supplementary Figure 14

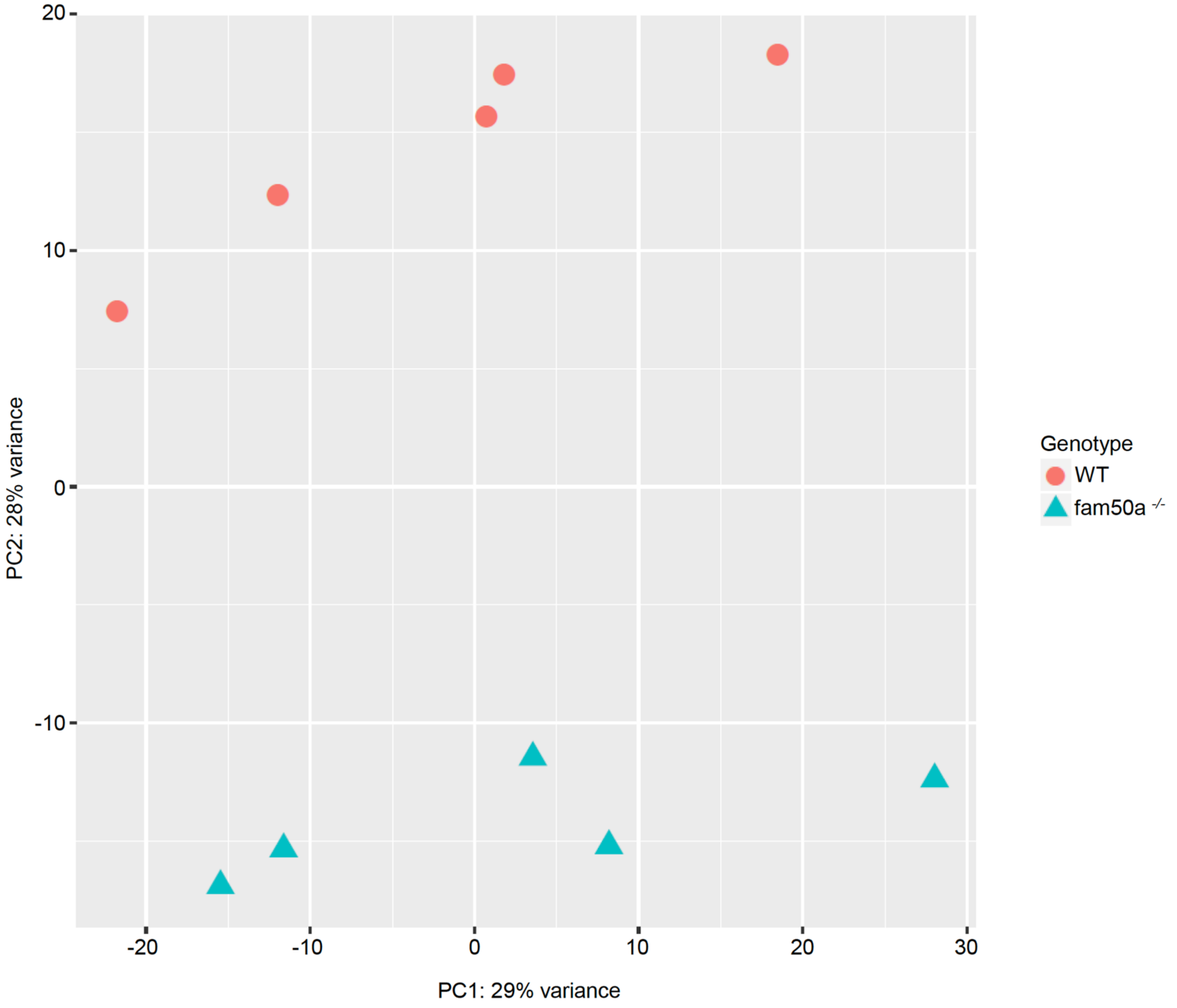

Supplementary Figure 14. Clustering analysis of RNA-seq data generated on *fam50a* KO zebrafish. Wild type and mutants are distributed into two distinct groups by principal component analysis (PCA). Samples separate out by genotype on PC2 (Y-axis), which explains 28% of the variance in the dataset. Wild type is shown with red circles, *fam50a* homozygous mutants are shown in green triangles. See NCBI Gene Expression Omnibus under accession number GEO: GSE145711 for source data.

Supplementary Figure 15

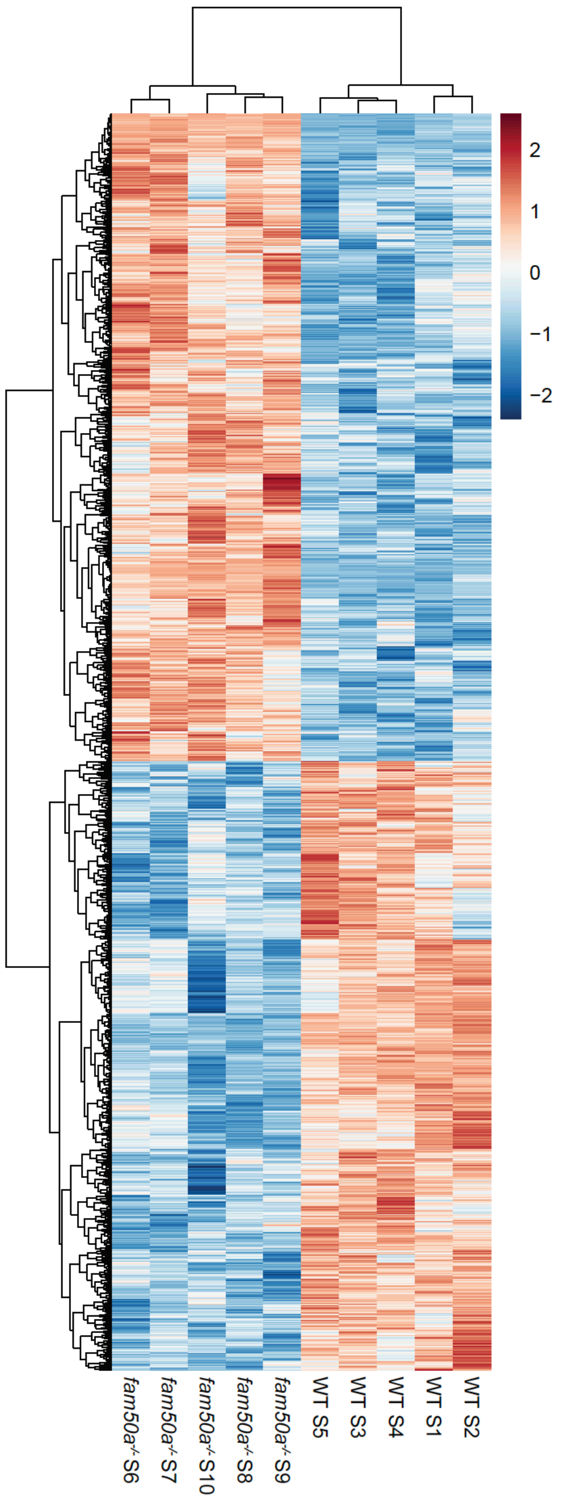

Supplementary Figure 15. Heat map of differentially expressed genes in WT vs. *fam50a* KO.
Heat map of transcriptome data corresponding to five replicates of each WT and *fam50a* KO zebrafish sequencing library. Statistical differences were calculated using a Wald test, FDR corrected using the Benjamini-Hochberg method; only genes with p-value <0.05 and >2-fold-change are included for each WT vs *fam50a* KO comparison. Gene expression has been z-score normalized and the samples and genes are clustered by correlation distance with complete linkage. NCBI Gene Expression Omnibus under accession number GEO: GSE145711.

Supplementary Figure 16

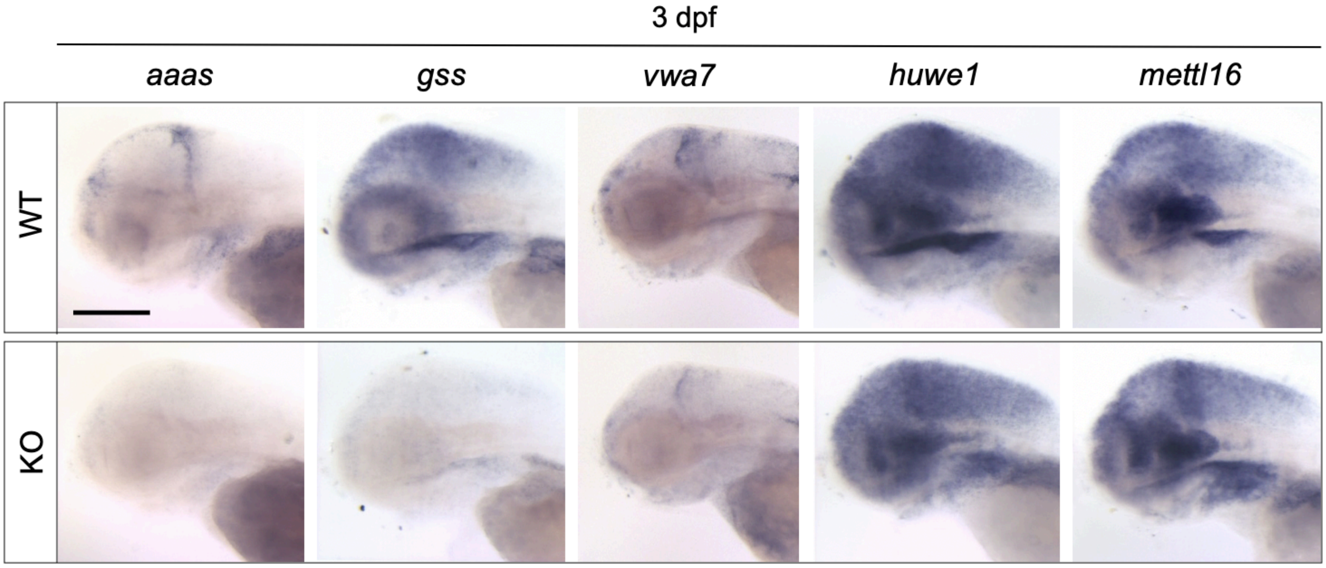

Supplementary Figure 16. Validation of additional genes whose transcript levels were altered in RNAseq analysis of *fam50a* KO zebrafish. Representative wholemount *in situ* hybridization (WISH) images. The transcript levels of *aaas*, *gss* and *vwa7* show reduced expression; and *mettl16* shows increased expression and were concordant with RNA-seq analysis. *huwe1* did not show significant differences between WT and *fam50a* KO according to RNA-seq but show modest reduction in expression according to WISH. See Table 2 for summary data. Number of larvae evaluated with similar results: *aaas*, n=11; *gss*, n=10; *vwa7*, n=11; *huwe1*, n=10; *mettl16*, n=10. Scale bar: 200 μm, with equivalent sizing across panels.

Supplementary Figure 17

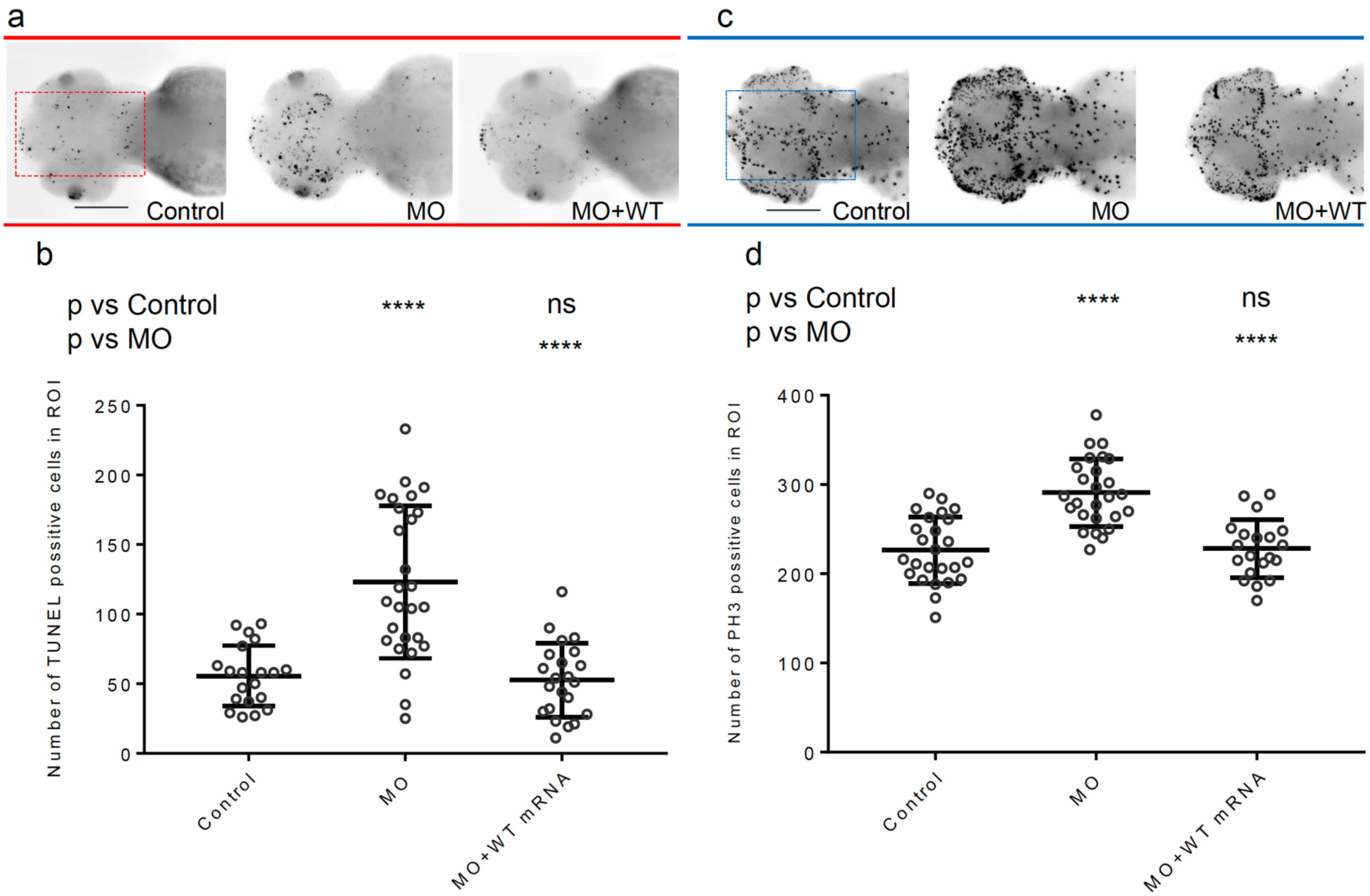

Supplementary Figure 17. *fam50a* suppression in zebrafish results in apoptosis and altered cell cycle progression.

a) Representative dorsal views of wholemount zebrafish heads stained with terminal deoxynucleotidyl transferase dUTP nick end labeling (TUNEL) show enrichment of apoptotic cells in morphants compared to controls; this defect was rescued by co-injection of human WT *FAM50A* mRNA. Scale bar, 200 μm, with equivalent sizing across panels. The region of interest (ROI) quantified is indicated with a red dotted rectangle.

b) Dot plot shows the distribution of TUNEL positive cells in the ROI (see a). TUNEL positive cells were quantified and the statistical differences were calculated using an unpaired student's t-test (two-sided); Exact p-values and number of larvae are listed in Supplementary Table 4; **** indicates p<0.0001; ns, not significant; data are presented as mean values +/- standard deviation.

c) Representative dorsal images of wholemount zebrafish heads marked with phospho-histone H3 (PH3) immunostaining. Scale bar, 200 μm, with equivalent sizing across panels. The ROI is highlighted with a blue dotted rectangle.

d) Dot plot showing quantification of PH3 positive cells in ROI. The statistical differences were calculated using an unpaired student's t-test (two-sided); Exact p-values and number of larvae are listed in Supplementary Table 4; **** indicates p<0.0001; ns, not significant; data are presented as mean values +/- standard deviation.

Supplementary Figure 18

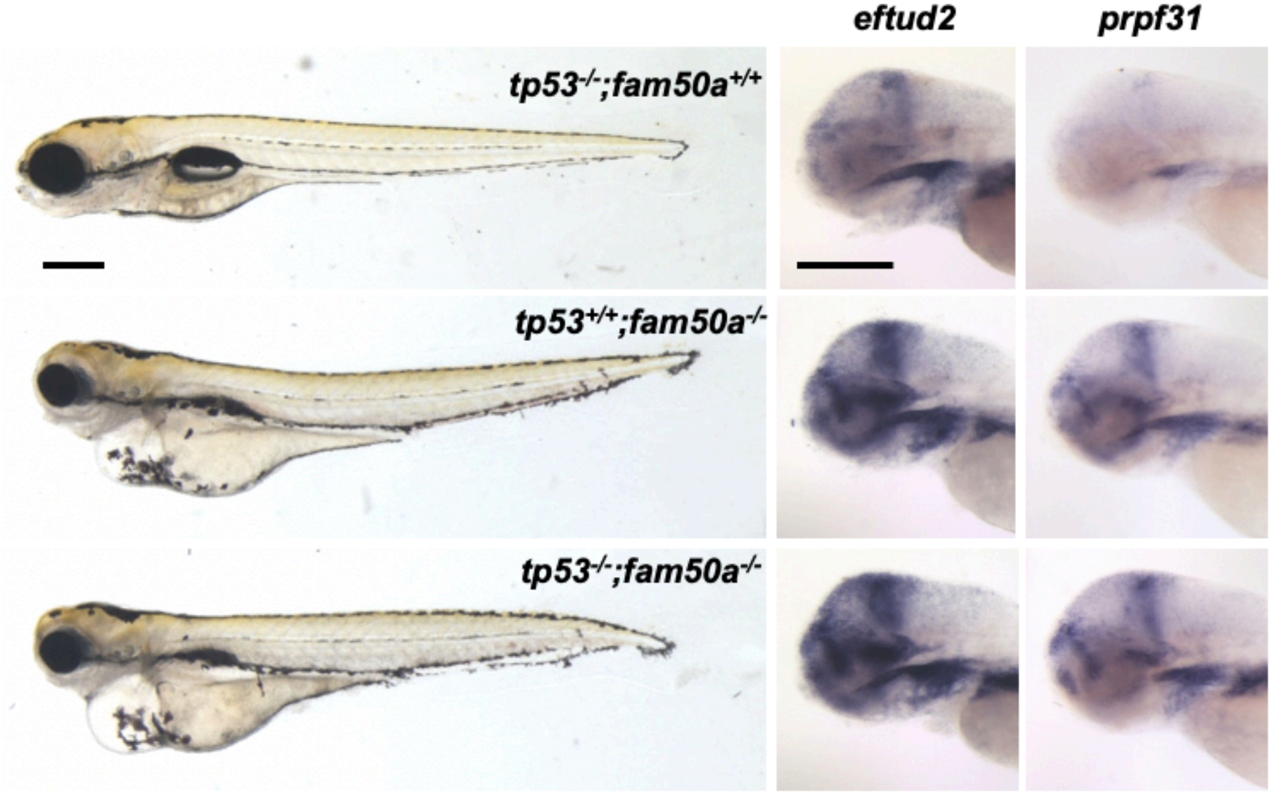

Supplementary Figure 18. Phenotypic analysis of *fam50a* KO zebrafish in a *p53* (*tp53* in zebrafish) KO background.

Similar phenotypes were observed between *fam50a* single KO zebrafish and *fam50a*/*tp53* double KO zebrafish at 5 dpf, n=182, repeated six times, with similar results. As representative molecular markers, *eftud2* and *prpf31* expression were examined in *fam50a* single KO zebrafish and *fam50a*/*tp53* double KO zebrafish at 3 dpf. *eftud2*, n=32, experiment repeated two times; *prpf31*, n=32, experiment performed once; all animals assessed showed similar results within each genotype. Scale bars; 200 μm, with equivalent sizing across panels.

Supplementary Figure 19

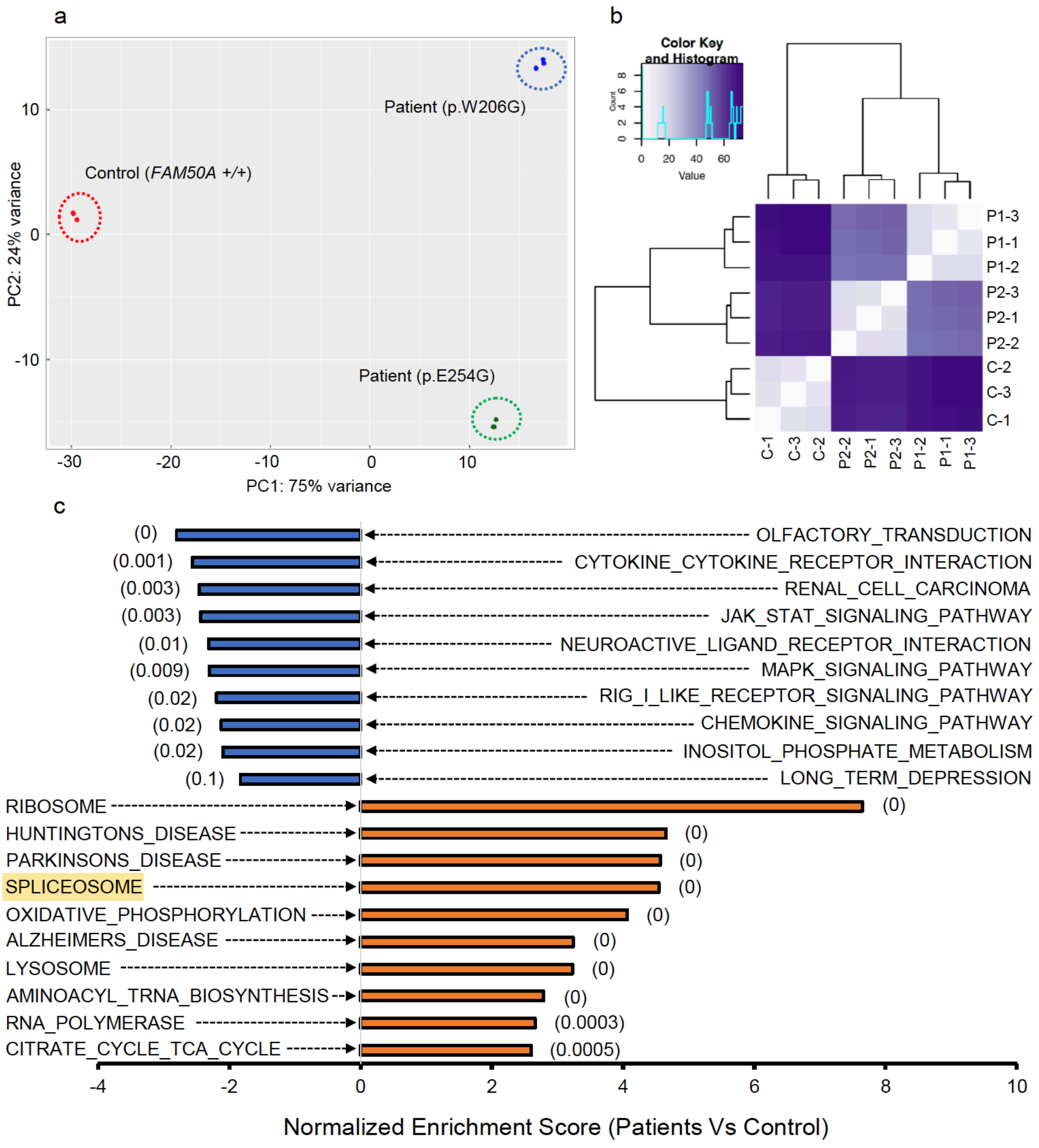

Supplementary Figure 19. RNA-seq data generated from FAM50A patient lymphocyte cell lines.

RNA-seq data from human lymphocyte cell line show alteration of overlapping functional groups compared to transcriptomics data generated on *fam50a* KO zebrafish.

a) Principal component analysis demonstrates independent clustering of *FAM50A* cases and male control sample of European origin. Samples from control (red circles) and affected individuals (green or blue circles) are separated on PC1 (X-axis), which explains 75% of the variance in the dataset.

b) Clustered heat map data generated on human lymphoblast cell transcriptomes. A correlation among *FAM50A* patients and control samples is shown using differential expression profiles. The color scale indicates the degree of correlation: white, high correlation; indigo, low correlation, as shown in the color key.

c) KEGG pathway enrichment analysis using normalized enrichment score for *FAM50A* cases vs control. The top ten significantly dysregulated pathways are plotted along the x-axis both for depleted and augmented gene sets. Down-regulated gene sets, orange; upregulated gene sets, blue. Spliceosome in the upregulated pathway is highlighted in yellow. Statistical differences were calculated using a Kolmogorov-Smirnov test; P-values (FDR corrected) are indicated in parentheses. See NCBI Gene Expression Omnibus under accession number GEO: GSE145710 for source data.

Supplementary Tables

Supplementary Table 1. Bioinformatic analyses of patient-derived missense mutations in *FAM50A*

In silico tool / database	p.Trp206Gly	p.Glu254Gly	p.Asp255Gly	p.Asp255Asn	p.Arg273Trp
iP Tree	Negative, Destabilizing	Negative, Destabilizing	Negative, Destabilizing	Negative, Destabilizing	Negative, Destabilizing
SIFT	Damaging	Tolerated	Tolerated	Tolerated	Damaging
Mutation Taster	Disease causing	Disease causing	Disease causing	Disease causing	Disease causing
I-Mutant	Decreased stability	Decreased stability	Decreased stability	Decreased stability	Decreased stability
PolyPhen	Probably damaging	Probably damaging	Probably damaging	Probably damaging	Probably damaging
Consurf	Highly conserved, buried	Highly conserved, exposed	Highly conserved, exposed	Highly conserved, exposed	Highly conserved, exposed
CADD	29	33	19	32	33
ExAC	Not found	Not found	Not found	Not found	Not found
gnomAD	Not found	Not found	Not found	Not found	Not found

iP Tree: http://203.64.84.190:8080/IPTREEr/iptree.htm
SIFT: https://sift.bii.a-star.edu.sg/
Mutation Taster: http://www.mutationtaster.org/
I-Mutant: http://gpcr2.biocomp.unibo.it/cgi/predictors/I-Mutant3.0/I-Mutant3.0.cgi
PolyPhen: http://genetics.bwh.harvard.edu/pph2/
Consurf: http://consurf.tau.ac.il/2016/
CADD: https://cadd.gs.washington.edu/
ExAC: http://exac.broadinstitute.org/
gnomAD: https://gnomad.broadinstitute.org/

Supplementary Table 2. Predicted binding free energy changes in the context of FAM50A missense variants

In silico tool	p.Trp206Gly	p.Glu254Gly	p.Asp255Gly	p.Asp255Asn	p.Arg273Trp
mCSM	-1.88	-1.06	-0.25	-0.92	-0.62
SDM	-4.46	-2.40	2.76	0.48	-0.20
DUET	-1.75	-1.32	-0.07	-0.89	-0.69
SAAFEC	0.72	8.15	2.29	1.46	1.60
FOLDX	0.70	-1.70	-4.20	-1.07	0.90

$\Delta\Delta G > 0$ indicates that mutations stabilize the protein. Energy changes are given in kcal/mol.
Related to Supplementary Figure 2.
mCSM: http://biosig.unimelb.edu.au/mcsm/
SDM: http://marid.bioc.cam.ac.uk/sdm2/
DUET: http://biosig.unimelb.edu.au/duet/
SAAFEC: http://compbio.clemson.edu/SAAFEC/
FOLDX: http://foldxsuite.crg.eu/

Supplementary Table 3. List of primer sequences.

Gene/Primer name	Primer	Nucleotide sequence (5' to 3')
Primer sequences used for wholemount RNA *in situ* probe synthesis		
fam50a	forward	AGAACCCAGACGTGGACACC
fam50a	reverse	AGTGTTCTGCTGCCCAAGGTC
cdkn1a	forward	GCGATCTGGAGGACGCATCG
cdkn1a	reverse	GCTACGTTTTGTGCAATAAGGCA
mdm2	forward	GAAACACACTCCAACTGGGA
mdm2	reverse	TCAATACAGCACCTCACAAGC
aaas	forward	TTAGGGCTTCCGTCAGTCTG
aaas	reverse	GGAGGAAGACCATCTCAGCA
eftud2	forward	CTTTGTGGAGGTTCAAGCACC
eftud2	reverse	GACACTAGACTGCAAGGGGA
gss	forward	CAGAGCTGGGAGGTGCGTTTGAT
gss	reverse	CCCGATGTCAGTCATACGCGT
huwe1	forward	GGGCTTTTATGAGATCATCCCCA
huwe1	reverse	GTTCGGTGTGTCAATGAGAGGCT
mettl16	forward	CGCCCTGTAAGAAGCGTAAGCT
mettl16	reverse	TCGGTTTTCCAGCATTGTGG
ice1	forward	TTTCCAAAAGCATGATCGCCA
ice1	reverse	ACTGTAACCGATGGGCAGAG
prpf3	forward	CCAGCTGTACCTCACTGGCA
prpf3	reverse	CAACAATGCCTGCCGTACAT
edar	forward	TGCTGTTCTGGTCAGCTCAT
edar	reverse	TCTCGTCTCTCTTCAGCCCG
prpf4	forward	CACTCATGGCGACTTTGGGA
prpf4	reverse	AACGCCCTGTACATCAAGACCA
snrnp200	forward	GCTCTCGTATGAAGTGGTTGAC
snrnp200	reverse	TCCTGTGGATTCACTACGCA
prpf6	forward	CGCCTCAAGAATCCACAAAGC
prpf6	reverse	ACAACGGAGACAACCACTCC
snapc4	forward	ATGGGAAAGGTGGAATCCCG
snapc4	reverse	CCCTTAAACTCCACAGAGTGC
prpf8	forward	AAGGAAATGGAGCCTCTTGG
prpf8	reverse	ACAATGTGCTTTCAGACTTGTGC
snrpe	forward	TGCGTCATTCTGTTTCCGGA
snrpe	reverse	TTGGACACACTCTGCAGCAACG
prpf31	forward	GAGAGCGCAGATGGCAAGGTTGG
prpf31	reverse	ATGTCACCGTCCCCACACAAGTC
txnl4a	forward	CGCCAAATTTAGCATACGAGACC
txnl4a	reverse	AGTCTTTAGGGGACACCACAA
sf3b4	forward	TGCTTTCCCACCTGTTCCTC
sf3b4	reverse	TTGGTCTTCACATGGGGGTT
eif4a3	forward	CTGGTGTTGGATGAAGCCGA
eif4a3	reverse	ATGATGAGGGAAACCTGCGA
vwa7	forward	GGCAGAAGATTCACCAGACTC
vwa7	reverse	AAGACAGACGCTCCAGCACAT
eda	forward	TCTCGATCCACCAGAGAGTG
eda	reverse	AGCGTAAGAACGGAGTTTTGTC
Primer sequences used for FAM50A human construct sequencing		

FAM50A Ex1 seq	forward	ATGGCTCAATACAAGGGCGC
FAM50A Ex4 seq	forward	AAGCCAAGCGGAAGATCTCC
FAM50A Ex8 seq	forward	CGCTCGAGATCCTTCGGAAA
Primer sequences used for site-directed mutagenesis		
FAM50A p.A137V F	forward	AGGAGGAAGAGGAGGTGGCCATGTATGAGGA
FAM50A p.A137V R	reverse	TCCTCATACATGGCCACCTCCTCTTCCTCCT
FAM50A p.E143K F	forward	GCCATGTATGAGGAGAAGATGGAAAGGGAAG
FAM50A p.E143K R	reverse	CTTCCCTTTCCATCTTCTCCTCATACATGGC
FAM50A p.W206G F	forward	ATCACCTTCAGCTACGGGGATGGCTCTGGGC
FAM50A p.W206G R	reverse	GCCCAGAGCCATCCCCGTAGCTGAAGGTGAT
FAM50A p.E254G F	forward	TCATGTACATCAAGGGGGACTTGATCATCCC
FAM50A p.E254G R	reverse	GGGATGATCAAGTCCCCCTTGATGTACATGA
FAM50A p.D255G F	forward	ATGTACATCAAGGAGGGCTTGATCATCCCTC
FAM50A p.D255G R	reverse	GAGGGATGATCAAGCCCTCCTTGATGTACAT
FAM50A p.D255N F	forward	ATGTACATCAAGGAGAACTTGATCATCCCTC
FAM50A p.D255N R	reverse	GAGGGATGATCAAGTTCTCCTTGATGTACAT
FAM50A p.R273W F	forward	ATCGTCACCAAGGCATGGGGGAAGAGTGGAC
FAM50A p.R273W R	reverse	GTCCACTCTTCCCCCATGCCTTGGTGACGAT
Primer sequences used for *FAM50A* tissue expression analysis		
cDNA-F	forward	ATGGCTCAATACAAGGGCGCCGCG
cDNA-R	reverse	GCGGATCGTGTACTTGTCCCACTTCTT
B-Actin-RT-F	forward	ACCTTCTACAATGAGCTGCG
B-Actin-RT-R	reverse	CCTGGATAGCAACGTACATGG
Primer sequences used for subcellular localization of tagged WT and mutant FAM50A		
FAM50A-F1	forward	CACCATGGCTCAATACAAGGGCGCCGCG
FAM50A-R1	reverse	GCGGATCGTGTACTTGTCCCACTTCTT
T7-F	forward	TAATACGACTCACTATAGGG
BGH-F	reverse	TAGAAGGCACAGTCGAGG
Primer sequences used for generation of *fam50a* CRISPR knockout zebrafish model		
sgRNA1-F	forward	AGAAAGGTTTGGAACAGCTTT
sgRNA2-R	reverse	ACATTAGAACCCATCCGACT
sgRNA-seq-F	forward	GTTTCAGGAAGAGGAAAATCGCT
sgRNA-seq-R	reverse	CGACTTATTTGCACATGCAGTG
KO-seq-F	forward	TTCAGCTACTGGGATGGCTC
KO-seq-R	reverse	CAACAATATGGCATTTTGGGTGC
Primer sequences used for transient suppression of *fam50a* in zebrafish		
sb-MO	morpholino	AGTAAAATCCCGTACATATCTCCTC
MO-seqRT-F	forward	AACAGAAGATCGCAGAGGACA
MO-seqRT-R	reverse	GAGAAACTGCTGGATTGTGTTG
Primer used for sequence confirmation of *EFTUD2* and *DDX41* Co-IP constructs		
EFTUD2-F1	forward	ACCGACTTATATGATGAGTTTGGGA
EFTUD2-F2	forward	CTCAGCCTCTCACAGAACCC
EFTUD2-F3	forward	GGCTGATCCTGGAGCTGAAG
EFTUD2-F4	forward	TTGGCGAGTTCACAGGCTTT
EFTUD2-F5	forward	AGATTGCTGTGGAGCCAGTC
EFTUD2-F6	forward	TCTGAGGTGGACAAGGCTCT
EFTUD2-F7	forward	CTGGCCCGGGAATTCATGAT
EFTUD2-R1	reverse	CCAGGGTGGTCATCGTCATG
DDX41-F1	forward	GAACCCGAACGGAAGCGG
DDX41-F2	forward	CTCAGTCCAACGTCAGCCTC
DDX41-F3	forward	TACCCTTCTCAAAGCGCGAG

DDX41-F4	forward	GCCAAGATGGTGTACCTGCT
DDX41-F5	forward	CCTGGGTCATCGGATCACTG
DDX41-R1	reverse	GGCACATAGGGCACGTAGTC

Supplementary Table 4. Statistical differences in stable and transient *fam50a* zebrafish models.

	N	Mean	SD	SEM	P vs control	P vs FAM50A-WT mRNA	P vs morphants	P vs WT-rescue	Interpretation*
fam50a KO craniofacial phenotyping, related to Figure 3b and Supplementary Figure 10a									
WT	20	96.89	12.03	2.69					
fam50a$^{+/-}$	37	96.35	15.13	2.49	0.8905				
fam50a$^{-/-}$	24	138.44	14.39	2.94	5.2742E-13				
Morpholino dose curve (craniofacial), related to Supplementary Figure 11d									
Control	56	87.47	5.28	0.70					
MO 3ng	36	88.73	7.29	1.21	0.3395				
MO 6ng	48	115.54	8.31	1.20	1.5394E-38				
MO 9ng	41	125.77	15.41	2.40	4.3164E-31				
Variants tested (craniofacial), related to Figure 3c and Supplementary Figure 10b									
Control	40	87.90	5.46	0.86			6.1110E-16	0.1377	
MO 5ng	45	103.84	8.64	1.28	6.1110E-16			2.3657E-12	
MO+WT	39	89.93	6.49	1.04	0.1377		2.3657E-12		
MO+p.A137V	36	89.34	6.16	1.03	0.2851		9.8773E-13	0.6896	Benign
MO+p.E143K	49	92.43	6.16	0.88	0.0005		5.6586E-11	0.0680	Benign
MO+p.D255G	46	103.51	7.77	1.15	3.3923E-17		0.8473	3.2926E-13	Pathogenic
Variants tested (craniofacial), related to Figure 3c and Supplementary Figure 10c									
Control	61	90.20	5.34	0.68			6.1359E-21	0.0002	
MO 5ng	78	111.43	7.16	0.81	6.1359E-21			1.8623E-21	
MO+WT	43	95.11	7.69	1.17	0.0002		1.8623E-21		
MO+p.D255N	66	106.23	8.94	1.10	7.1448E-23		0.0002	1.0591E-09	Pathogenic
MO+p.R273W	69	100.49	7.60	0.91	7.1830E-15		1.3212E-15	0.0004	Pathogenic
Variants tested (craniofacial), related to Figure 3c and Supplementary Figure 10d									
Control	35	92.22	5.92	1.01			3.4646E-29	0.0615	
MO 5ng	41	124.05	7.14	1.11	3.4646E-29			1.6332E-26	
MO+WT	44	99.16	7.48	1.12			1.6332E-26		
MO+p.W206G	32	104.11	5.78	1.02	6.5824E-07		3.4999E-20	0.0025	Pathogenic
Variants tested (craniofacial), related to Figure 3c and Supplementary Figure 10e									
Control	38	90.14	6.76	1.09			2.4686E-30	0.0502	
MO 5ng	44	125.24	10.01	1.51	2.4686E-30			5.5137E-26	
MO+WT	33	93.26	6.38	1.11	0.0502		5.5137E-26		
MO+p.E254G	36	100.25	8.49	1.41	2.6495E-07		3.4505E-19	0.0003	Pathogenic
Overexpression (craniofacial), related to Supplementary Figure 12 (left)									
Control	44	87.21	7.05	1.06		0.0571			
WT	37	90.01	5.79	0.95	0.0571				
p.A137V	38	90.17	5.73	0.93	0.042	0.9043			
p.E143K	42	91.13	5.87	0.90	0.0065	0.3996			
p.D255G	53	90	7.45	1.02	0.0633	0.9913			
p.D255N	39	90.28	5.89	0.94	0.0356	0.8432			
Overexpression (craniofacial), related to Supplementary Figure 12 (right)									
Control	50	88.51	4.75	0.67		0.0049			
WT	42	91.36	4.64	0.72	0.0049				
p.W206G	48	92.78	5.10	0.74	4.4193E-05	0.1728			
p.E254G	36	93.86	4.28	0.71	7.2727E-07	0.0163			
p.R273W	49	93.08	4.71	0.67	5.7480E-06	0.0825			
Apoptosis phenotyping in *fam50a* transient knockdown zebrafish, related to Supplementary Figure 17b									
Control	20	55.65	21.7	4.85			4.8236E-06	0.6953	
MO 5ng	27	123.04	54.81	10.55	4.8236E-06			1.4718E-06	
MO+WT	22	52.68	26.52	5.66	0.6953		1.4718E-06		
Cell cycle phenotyping in *fam50a* transient knockdown zebrafish, related to Supplementary Figure 17d									
Control	25	226.44	37.19	7.44			1.4977E-07	0.8833	
MO 5ng	26	290.81	37.82	7.42	1.4977E-07			4.3573E-07	
MO+WT	20	228	32.56	7.28	0.8833		4.3573E-07		

P-values determined with an unpaired student's t-test (two-sided).

Supplementary Table 5. DAVID analysis showing enriched functional groups of genes with altered mRNA splicing in *fam50a*$^{-/-}$ vs. control zebrafish heads at 2 dpf. Related to Figure 4.

GO term*	Description	Fold enrichment**	P-value$^{\Psi}$	Bonferroni^	Genes
**Alternative 3' splicing events**					
GO:0005634	nucleus	1.95	2.20E-07	2.25E-05	qkia, kat6b, kdm3b, eya3, atf7ip, adrm1, prpf39, zc3h18, mier1a, ebf3a, u2af2b, ilf3b, ccnl1a, pou2f1b, sim1b, tcf7l2, arnt2, ep300b, nav3, smarcc1b, tcf3a, elf2a, eya4, rnf111, chd4a, rps6ka3a, stat5b, phf20l1, foxp1a, mdm4, arnt, chd1, otud7b, dot1l, hdac9b, mcmbp, tnfrsf1b, psen1, rtfdc1, hmga1a, mvp, rbm39a, rbmx, tdg.2, foxp1b, ggnbp2, akap8l, smg1, ep300a, apex1, mef2aa, tfdp2, etv5a, nfyc
GO:0003677	DNA binding	2.58	1.125E-06	0.00018	chd4a, kat6b, stat5b, hipk2, foxp1a, pogzb, si:ch211-266o15.1, arnt, chd1, otud7b, csde1, ZNF335, ybx1, hmga1a, zfand5a, mier1a, ebf3a, foxp1b, ilf3b, pou2f1b, lef1, sim1b, rfc4, zgc:77486, arnt2, akap8l, smarcc1b, apex1, mef2aa, tfdp2, elf2a, etv5a
GO:0003723	RNA binding	3.68	1.67E-06	0.00026	qkia, elavl3, ybx1, ewsr1a, ankhd1, rbm17, pcbp3, rbm39a, hug, u2af2b, rbmx, rpl22l1, ilf3b, elavl4, cirbpb, ilf3a, igf2bp2a, eif4g1a, apex1, ewsr1b
GO:0006355	regulation of transcription, DNA-templated	2.53	6.06E-06	0.0024	chd4a, kat6b, stat5b, phf20l1, foxp1a, kdm3b, eya3, arnt, csde1, atf7ip, sap130a, hdac9b, ybx1, hmga1a, ebf3a, foxp1b, ccnl1a, ilf3b, pou2f1b, sim1b, arnt2, ep300a, ep300b, mef2aa, tfdp2, elf2a, etv5a, eya4, nfyc
GO:0003676	nucleic acid binding	2.20	7.87E-05	0.012	qkia, elavl3, chd4a, ankhd1, rbm17, pcbp3, si:dkey-217d24.6, phf20l1, hnrnpaba, foxp1a, zcchc8, pogzb, csde1, igf2bp2a, rbm33a, ZNF335, celf5a, si:dkey-205h23.2, ybx1, ewsr1a, rbm39a, hug, rbmx, u2af2b, foxp1b, elavl4, cirbpb, tardbpl, ewsr1b
GO:0005667	transcription factor complex	7.32	0.0001	0.01	lef1, sim1b, eya3, tcf7l2, arnt2, arnt, tcf3a, tfdp2
GO:0007420	brain development	5.04	0.001	0.33	arnt2, psen1, kdm6al, mib1, fam57ba, mvp, ldlrb, tnfrsf1b
GO:0006509	membrane protein ectodomain proteolysis	34.01	0.0031	0.71	adam10a, psen1, rbmx
GO:0006351	transcription, DNA-templated	2.28	0.0039	0.79	stat5b, ebf3a, rbmx, foxp1b, ilf3b, pou2f1b, foxp1a, sim1b, eya3, arnt2, arnt, atf7ip, hdac9b, mef2aa, tfdp2, eya4
GO:0019013	viral nucleocapsid	10.63	0.006	0.46	elavl3, hug, hnrnpaba, elavl4
GO:0004402	histone acetyltransferase activity	10.57	0.006	0.62	kat6b, ep300a, sap130a, ep300b
GO:0019012	virion	9.98	0.0071	0.52	elavl3, hug, hnrnpaba, elavl4
GO:0000981	RNA polymerase II transcription factor activity, sequence-specific DNA binding	4.032	0.0076	0.70	sim1b, si:ch211-266o15.1, tfdp2, elf2a, etv5a, foxp1b, foxp1a
GO:0006397	mRNA processing	4.62	0.0093	0.97	qkia, srrm1, rbm39a, rbmx, u2af2b, prpf39

dre04520	Adherens junction	5.62	0.01	0.39	lef1, tcf7l2, ep300a, ep300b, vcla
dre04916	Melanogenesis	5.55	0.01	0.40	lef1, tcf7l2, ep300a, ep300b, adcy7
GO:0060070	canonical Wnt signaling pathway	8.14	0.012	0.99	lef1, tcf7l2, psen1, dot1l
GO:0007219	Notch signaling pathway	7.94	0.013	0.99	adam10a, psen1, otud7b, mib1
GO:0046983	protein dimerization activity	4.22	0.013	0.88	sim1b, arnt2, arnt, tcf3a, usf1, mef2aa
GO:0010628	positive regulation of gene expression	14.88	0.017	0.998	lef1, apex1, mef2aa
dre04512	ECM-receptor interaction	6.78	0.019	0.60	thbs1b, hmmr, itgb4, hspg2
GO:0003700	transcription factor activity, sequence-specific DNA binding	2.10	0.019	0.95	stat5b, hmga1a, foxp1b, foxp1a, pou2f1b, sim1b, arnt, arnt2, tcf3a, elf2a, tfdp2, etv5a, nfyc
GO:0005794	Golgi apparatus	2.62	0.032	0.96	st6galnac6, psen1, clasp2, nav3, gnptab, rnf144ab, si:ch211-220f16.2, gosr1
GO:0008270	zinc ion binding	1.64	0.032	0.99	rnf111, nup153, kat6b, chd4a, ewsr1a, zfand5a, mib1, phf20l1, zcchc8, zgc:77486, si:ch211-266o15.1, mdm4, otud7b, ep300a, ep300b, micall2a, rnf144ab, ewsr1b, scaf11, rsf1b.1
dre04310	Wnt signaling pathway	3.87	0.036	0.82	lef1, tcf7l2, psen1, ep300a, ep300b
GO:0021979	hypothalamus cell differentiation	52.91	0.037	0.99	lef1, arnt2
GO:0016576	histone dephosphorylation	52.91	0.037	0.99	eya3, eya4
GO:0048025	negative regulation of mRNA splicing, via spliceosome	52.91	0.037	0.99	ybx1, rbmx
GO:0007275	multicellular organism development	2.19	0.038	0.99	qkia, si:ch211-266o15.1, eya3, arnt2, mib1, ccdc85ca, ebf3a, ncl1, rbmx, eya4
GO:0051010	microtubule plus-end binding	51.07	0.038	0.99	clasp2, clasp1a
GO:0043515	kinetochore binding	51.07	0.038	0.99	cenpe, clasp1a
GO:2001240	negative regulation of extrinsic apoptotic signaling pathway in absence of ligand	39.69	0.049	0.99	lef1, tcf7l2, psen1, ep300a, ep300b
**Alternative 5' splicing events**					

GO:0003676	nucleic acid binding	5.026	0.0029	0.06	cpsf6, syncrip, ddx3b, taf15, trnau1apb, celf2
GO:0000166	nucleotide binding	3.83	0.0096	0.18	cpsf6, syncrip, ddx3b, taf15, trnau1apb, celf2
**Intron retention events**					
GO:0006412	translation	4.718	0.019	0.89	rps2, gfm2, qrsl1, rpl10a, rpl13a
GO:0015934	large ribosomal subunit	55.478	0.034	0.86	rpl10a, rpl13a
GO:0003723	RNA binding	3.76	0.039	0.99	rps2, ythdc1, rae1, rpl10a, parn
**Exon skipping events**					
GO:0003723	RNA binding	4.079	0.00029	0.02	eif4bb, pcbp3, rpl9, u2af2a, sf1, eif4g1a, u2af2b, celf2, pcbp2, hnrpl, rbm4.1
GO:0000166	nucleotide binding	1.98	0.008	0.51	srsf3a, MARK4, u2af2a, rps24, ntrk2a, u2af2b, celf2, atp11c, gfm2, eif4bb, gnal, hnrnph3, rps6kb1b, myo5b, pak6b, hnrpl, rbm4.1
GO:0003676	nucleic acid binding	2.15	0.01	0.60	srsf3a, pcbp3, u2af2a, sf1, u2af2b, celf2, pcbp2, pogzb, eif4bb, npm1b, hnrnph3, chd4b, hnrpl, rbm4.1
GO:0008187	poly-pyrimidine tract binding	154.29	0.013	0.68	u2af2a, u2af2b
GO:0089701	U2AF	89.33	0.022	0.75	u2af2a, u2af2b
GO:0006397	mRNA processing	6.26	0.025	0.99	u2af2a, u2af2b, celf2, hnrpl
GO:0030628	pre-mRNA 3'-splice site binding	77.15	0.025	0.90	u2af2a, u2af2b
GO:0070507	regulation of microtubule cytoskeleton organization	53.72	0.036	0.99	traf3ip1, atat1
GO:0000243	commitment complex	39.70	0.048	0.96	u2af2a, u2af2b

*GO, Gene ontology, http://geneontology.org/ ; dre, KEGG pathway, https://www.genome.jp/kegg/pathway.html

** Proportion of genes found over hits found in background (percent genes found from query / percent hits found in background)

$^{\Psi}$ DAVID derived p-value (Fisher's exact test)

^Adjusted p-value

Supplementary Table 6. DAVID analysis showing enriched functional groups of genes with altered mRNA splicing in cases vs. control lymphoblast cell lines. Related to Supplementary Figure 19.

GO term*	Description	Fold enrichment**	P-value$^{\Psi}$	Bonferroni^	Genes
**Alternative 3' splicing events**					
GO:0016020	membrane	2.001879	3.56E-04	0.069347808	LST1, RPL13, OFD1, CSE1L, CLK3, ATIC, PSMD2, ZNF106, HNRNPC, ACSL4, CLINT1, SLC30A6, CHD4, RHEBL1, FMR1, STRN4, HLA-A, BCL2L12, IL11RA, HLA-DQA1, ELMO2, EIF4G1, VEGFB, PITPNM1, MPHOSPH9, NPC1, ATP13A1, DPM1, PDCD6IP
GO:0005654	nucleoplasm	1.800144	7.54E-04	0.141409389	ING4, DBF4B, LITAF, FAM193B, MKNK2, PPP6R3, TRRAP, HMGN5, SF3B2, ISG20, PLAGL1, MPPE1, TRIM66, CSE1L, CLK3, PSMD2, PBRM1, CC2D1B, HNRNPC, CLINT1, CHD4, TRMU, C17ORF49, SMAD7, SREK1, DDX39B, FMR1, BANP, UPF3A, WDR61, MAPK9, RNPC3, HDAC7
GO:0005515	protein binding	1.286617	0.001063	0.216976217	DBF4B, RPL13, PHF23, GGT1, ZNF346, OFD1, CLK3, PSMD2, IGFLR1, QKI, INO80E, CLINT1, SIK3, STRN4, FMR1, DDX39B, HLA-A, BANP, TECR, FAM118B, ELMO2, VEGFB, WDR48, EIF4G1, TARBP2, PITPNM1, NPC1, NOP2, MIB2, MAPK9, PDCD6IP, MTFR1L, SEPT6, ABLIM1, ING4, HAX1, LITAF, CEP95, TMEM218, MKNK2, GCSAM, PPP6R3, TRRAP, TJAP1, SF3B2, MPPE1, CSE1L, CEP170, GMPPA, ENO2, CD22, PBRM1, CC2D1B, HNRNPC, C19ORF60, CHD4, RHEBL1, UNC45A, UBXN11, SMAD7, MSH5, SREK1, KIF18B, HDDC3, ZBTB44, NAT9, WSB1, UPF3A, FNBP1, APOL1, WDR61, ZSCAN32, DPM1, SETD6, GGA2, GGA3, HDAC7
GO:0008380	RNA splicing	6.496297	7.11E-04	0.317772043	SREK1, FMR1, DDX39B, QKI, RNPC3, HNRNPC, SF3B2
GO:0070971	endoplasmic reticulum exit site	35.04615	0.003146	0.470901601	MPPE1, HLA-A, PDCD6IP
GO:0044822	poly(A) RNA binding	2.080302	0.008931	0.87297277	RBM33, RPL13, SREK1, FMR1, DDX39B, HLA-A, ZNF346, HMGN5, SF3B2, EIF4G1, NOP2, CLK3, QKI, USP36, ZNF106, HNRNPC
GO:0005794	Golgi apparatus	2.287679	0.010773	0.887864053	PLAGL1, ARFGAP2, MPHOSPH9, NPC1, MPPE1, LST1, LITAF, TANGO2, HLA-A, TRRAP, GGA2, CLINT1, SLC30A6
GO:0045727	positive regulation of translation	11.6268	0.004799	0.924845207	UPF3A, TARBP2, FMR1, DDX39B
hsa00230	Purine metabolism	4.158003	0.029332	0.957392715	ATIC, HDDC3, DGUOK, GUK1, AMPD3
GO:0005737	cytoplasm	1.337776	0.019026	0.979355995	ABLIM1, ARFGAP2, LST1, DBF4B, LITAF, CEP95, FAM193B, MKNK2, PHF23, GCSAM, PPP6R3, ZNF346, ISG20, MPPE1, CSE1L, CEP170, GMPPA, PHTF1, QKI, ACSL4, CHD4, SIK3, RHEBL1, UBXN11, C17ORF49, SMAD7, DDX39B, STRN4, FMR1, KIF18B, ANKRD13A, TECR, ELMO2, P2RX5, PANK4, EIF4G1, UPF3A, TARBP2, MPHOSPH9, PITPNM1, RPAIN, WDR61, MIB2, SETD6, PDCD6IP, HDAC7
**Alternative 5' splicing events**					
GO:0044822	poly(A) RNA binding	3.858625	8.32E-06	0.00121342	ZC3H14, HMGN2, RBM3, MRPS11, HLA-A, CNOT1, DDX5, RPS3, SAFB2, R3HDM1, PPIE, SRSF5, HNRNPH3, CLK3, RPLP0, PLEC
GO:0005634	nucleus	1.656845	0.001217968	0.140255598	NDUFAF6, HMGN3, HMGN2, HP1BP3, RBM3, ZNF131, PML, RABGAP1L, CNOT1, MXI1, RPS3, ACTG1, SFSWAP, CLK3, UFM1, ACTR6, RPLP0, PSMC3IP, PQBP1, C7ORF49, CREB3L4, ZC3H14, SDR39U1, ESPL1, DDX5, TACC1, SAFB2, TARBP2, PPIE, SRSF5, HNRNPH3, PARP2
GO:0003723	RNA binding	3.982072	0.003464605	0.397525924	PPIE, SFSWAP, SRSF5, HNRNPH3, COA1, RBM3, RNASET2, RPS3

hsa03040	Spliceosome	9.403964	0.007283585	0.422050051	PPIE, SRSF5, PQBP1, DDX5
GO:0005737	cytoplasm	1.55701	0.007004731	0.581736251	NDUFAF6, HMGN3, HMGN2, COA1, RBM3, PML, CNOT1, MXI1, UBQLN1, RPS3, ST6GALNAC6, UFM1, ACTR6, RPLP0, PQBP1, C7ORF49, GSTZ1, GSKIP, PCMT1, PLEC, ZC3H14, ESPL1, TACC1, SAFB2, TARBP2, SRSF5, C21ORF2, TBCD, PARP2
GO:0030529	intracellular ribonucleoprotein complex	8.246154	0.012159351	0.780631246	HNRNPH3, RPLP0, DDX5, RPS3
GO:0005515	protein binding	1.301709	0.011663251	0.819647637	HMGN2, HP1BP3, RBM3, TAF1D, PML, TMEM218, CNOT1, UBQLN1, NPRL2, MXI1, RPS3, ACTG1, SFSWAP, RASGRP3, CLK3, ACTR6, RPLP0, C7ORF49, PQBP1, GSTZ1, PCMT1, PLEC, ZC3H14, C14ORF159, PLXNB2, MSH5, HLA-A, ESPL1, BCS1L, DDX5, TACC1, SAFB2, TMEM106C, TARBP2, PPIE, SRSF5, HNRNPH3, C21ORF2, TBCD, ATG16L1, PARP2, SNX11
GO:0045111	intermediate filament cytoskeleton	16.17515	0.0143809	0.834068141	CLK3, TACC1, PLEC
GO:0042277	peptide binding	13.84445	0.019258097	0.941522231	RPLP0, NLN, ERAP2
GO:0005730	nucleolus	2.617216	0.030135907	0.977501724	ZC3H14, SRSF5, RBM3, PML, DDX5, MXI1, PARP2, RPS3
**Intron retention events**					
hsa04145	Phagosome	7.396774	5.78E-06	9.13E-04	CYBA, HLA-A, TUBA4A, SCARB1, HLA-C, HLA-B, HLA-DOA, HLA-DMA, ATP6V0B, HLA-F
hsa05332	Graft-versus-host disease	20.17302	9.10E-06	0.001437	HLA-A, HLA-C, HLA-B, HLA-DOA, HLA-DMA, HLA-F
hsa05330	Allograft rejection	17.99215	1.63E-05	0.002568	HLA-A, HLA-C, HLA-B, HLA-DOA, HLA-DMA, HLA-F
hsa04940	Type I diabetes mellitus	15.85023	3.07E-05	0.004838	HLA-A, HLA-C, HLA-B, HLA-DOA, HLA-DMA, HLA-F
GO:0005737	cytoplasm	1.569262	2.49E-05	0.005271	KIF22, HMGN2, NIT2, RBM3, RPS6KB2, ITSN2, MCM10, MAF1, DDX11, SPINT2, PRMT5, QKI, ZYX, EIF2B5, EXOSC8, RAN, DDX39B, TRIM41, PI4KA, MTA1, RRP8, MBD1, DDIT3, DAPK1, LILRB1, BBS2, PITPNM1, ARHGAP33, CAPN12, SPAG4, NEK8, CELF2, ZNF383, EEF1D, BIN2, UBE2T, LST1, ENDOV, ELMOD3, TUBGCP6, ATXN2L, TRIM69, NMRAL1, RASAL3, BDH2, SCARB1, RPL10A, BAZ2A, FGD3, SREBF1, SHMT2, MAP2K2, TONSL, KIF18B, RPS9, ILF3, SIRT7, U2AF1L4, PSMB9, NMT2, APOL3, ATF4, MRPL28, INVS, IRF7, HDAC6, ACTR10
GO:0002479	antigen processing and presentation of exogenous peptide antigen via MHC class I, TAP-dependent	13.42286	1.28E-05	0.009941	CYBA, PSMD13, HLA-A, HLA-C, HLA-B, PSMB9, HLA-F
hsa05320	Autoimmune thyroid disease	12.80211	8.76E-05	0.01375	HLA-A, HLA-C, HLA-B, HLA-DOA, HLA-DMA, HLA-F
GO:0042612	MHC class I protein complex	44.4759	8.26E-05	0.017352	HLA-A, HLA-C, HLA-B, HLA-F
hsa05416	Viral myocarditis	11.67912	1.36E-04	0.021332	HLA-A, HLA-C, HLA-B, HLA-DOA, HLA-DMA, HLA-F
GO:0030670	phagocytic vesicle membrane	12.43818	1.16E-04	0.024318	CYBA, HLA-A, HLA-C, HLA-B, ATP6V0B, HLA-F
**Exon skipping events**					
GO:0005737	cytoplasm	1.582606682	3.54E-06	8.21E-04	CAST, LDHA, SEC31A, AGTPBP1, NT5C3B, IFI44L, UBQLN1, NAP1L4, RPS3, WARS, MAX, CDCA7, CD44, HSF1, PCBP2, GSTZ1, ADAM8, MX1, CASP2, FANCA, USP15, ASPM, KIF13B, MRI1, SYNRG, OPA1, ARHGEF1, BRAF, INPPL1, NADSYN1, HCFC1R1, YTHDF1, MBNL1, PMM2, ISCU, TRNAU1AP, BPTF, VEGFA, CELF2,

					LRCH3, BIN1, CARM1, NEK6, USP3, CYTH1, HAUS2, FAM193B, MKNK2, CTNND1, VCPKMT, ZNF655, RCC1, BANF1, GCH1, STAT6, CHD1L, VRK3, MACF1, MSI2, KLHL42, RPS24, CHD3, HNRNPAB, TMC6, CRIP3, NASP, SMG7, MZB1, RPS9, ANKHD1, MPRIP, DRAM2, ANXA11, CD79B, TNK2, MYO19, EIF4E2, MPHOSPH8
GO:0005515	protein binding	1.308057272	2.43E-05	0.007633711	CAST, MPZL1, LDHA, SEC31A, NT5C3B, RBM5, UBQLN1, EPC1, PICALM, CSNK2A1, CD44, CLK2, SUPT20H, ADAM8, MX1, KIF13B, OPA1, RCOR3, BRAF, BCL2A1, YIF1A, ISCU, LAT2, CHID1, VEGFA, LRCH3, BIN1, NEK6, PAM, ALDOC, MTX2, CCDC14, RCC1, BANF1, VRK3, MACF1, HNRNPC, POLQ, HNRNPAB, NIN, SMG7, RPS9, MPRIP, POC1A, APOL1, ARMC8, CD79B, NUTF2, TNK2, EIF4E2, NCOR2, MLH3, NAP1L4, SCIMP, RPS3, WARS, MAX, HSF1, GRIPAP1, P4HA1, SLMAP, PCBP2, GSTZ1, IGFLR1, FANCA, CASP2, USP15, SYNRG, ARHGEF1, ARHGEF7, INPPL1, NADSYN1, CCNC, MBNL1, PMM2, MTMR14, BPTF, EIF4A2, SDCCAG3, CARM1, SNX10, MEGF8, EXOC7, CYTH1, MKNK2, BTN2A2, CTNND1, VCPKMT, ZNF655, ITM2C, GCH1, STAT6, CHD1L, KLC1, PIKFYVE, CD22, MSI2, KLHL42, CHD3, IL18R1, CPT1B, TMC6, MSH5, NASP, ANKHD1, PCK2, MON2, ANXA11, ZC3H11A, CENPU, MPHOSPH8, WDR20, SH3BP2
GO:0005829	cytosol	1.630053667	2.68E-04	0.06023605	CAST, LDHA, SEC31A, NT5C3B, AGTPBP1, GPCPD1, RPS3, WARS, CSNK2A1, HSF1, PCBP2, GSTZ1, MX1, CASP2, KIF13B, MRI1, ARHGEF1, BRAF, ARHGEF7, INPPL1, NADSYN1, DGUOK, PMM2, ISCU, MTMR14, TRAPPC9, EIF4A2, MGEA5, CARM1, NEK6, ATG10, EXOC7, PFKFB4, CYTH1, HAUS2, ALDOC, CTNND1, BANF1, GCH1, STAT6, KLC1, PIKFYVE, HNRNPC, RPS24, SMG7, RPS9, MON2, NUTF2, CENPU, PHPT1, EIF4E2
GO:0044822	poly(A) RNA binding	2.161759527	6.39E-04	0.182271325	CAST, ARHGEF1, RBM5, RPS9, MBNL1, YTHDF1, ANKHD1, NAP1L4, RPS3, TRNAU1AP, MACF1, EIF4A2, SRRM2, PCBP2, ANXA11, MSI2, CELF2, ZC3H11A, HNRNPC, ALG13, EIF4E2, CHD3, HNRNPAB, RPS24
GO:0005654	nucleoplasm	1.598436247	0.00182193	0.344969423	HAUS2, FAM193B, RBM5, MKNK2, IFI44L, UBQLN1, RCC1, BANF1, RPS3, GCH1, STAT6, MAX, EPC1, CHD1L, CDCA7, CSNK2A1, HSF1, CLK2, SRRM2, PCBP2, HNRNPC, POLQ, FANCA, HNRNPAB, CHD3, RPS24, OPA1, NASP, RPS9, CCNC, MBNL1, ANKHD1, BPTF, MSL1, ANXA11, CD79B, ZC3H11A, NUTF2, CENPU, CARM1, NEK6, NCOR2
hsa04910	Insulin signaling pathway	4.673233696	0.008365255	0.650081496	EXOC7, BRAF, INPPL1, MKNK2, PCK2, EIF4E2
GO:0030983	mismatched DNA binding	27.73439211	0.004928929	0.789116956	MSH5-SAPCD1, MSH5, MLH3
GO:0051225	spindle assembly	15.26198591	0.002174868	0.850540112	HAUS2, RCC1, NEK6, RPS3
GO:0000790	nuclear chromatin	3.842872635	0.009835961	0.899061082	STAT6, PAM, USP3, NASP, HNRNPC, RCC1, NCOR2
GO:0005634	nucleus	1.291399858	0.011879816	0.937501905	MEF2B, LDHA, AGTPBP1, RBM5, MLH3, NAP1L4, RPS3, WARS, MAX, EPC1, CSNK2A1, PICALM, CDCA7, HSF1, CLK2, PCBP2, MX1, CASP2, FANCA, USP15, ASPM, IZUMO4, SDR39U1, RCOR3, BRAF, MBD5, HCFC1R1, CCNC, MBNL1, DGUOK, ISCU, TRNAU1AP, ZNF195, CHID1, BPTF, MGEA5, CELF2, PRDM2, CARM1, NEK6, SNX10, MEGF8, USP3, FAM193B, MKNK2, CTNND1, RABGAP1L, ZNF655, RCC1, BANF1, GCH1, STAT6, CHD1L, VRK3, HNRNPC, RPS24, CHD3, HNRNPAB, SP140L, NASP, SMG7, RPS9, TNK2, CENPU, MPHOSPH8, NCOR2

*GO, Gene ontology, http://geneontology.org/ ; dre, KEGG pathway, https://www.genome.jp/kegg/pathway.html.

** Proportion of genes found over hits found in background (percent genes found from query / percent hits found in background)

$^{\Psi}$ DAVID derived p-value (Fisher's exact test)

^ p-value

Supplementary Table 7. Gene ontology enrichment analysis using proteins identified by mass spectrometry.

GO term*	Description	Observed	Expected	Fold enrichment	P value**
(GO:0030623)	U5 snRNA binding	2	0.01	> 100	1.33E-02
(GO:0033677)	DNA/RNA helicase activity	2	0.01	> 100	2.08E-02
(GO:0030620)	U2 snRNA binding	2	0.02	83.98	4.02E-02
(GO:0070180)	large ribosomal subunit rRNA binding	2	0.02	83.98	3.94E-02
(GO:0048027)	mRNA 5'-UTR binding	8	0.12	67.19	1.26E-09
(GO:0003735)	structural constituent of ribosome	32	0.83	38.61	4.90E-37
(GO:0017116)	single-stranded DNA helicase activity	3	0.09	34.99	1.32E-02
(GO:0008143)	poly(A) binding	3	0.1	31.49	1.64E-02
(GO:0016875)	ligase activity, forming carbon-oxygen bonds	6	0.2	29.99	2.00E-05
(GO:0004812)	aminoacyl-tRNA ligase activity	6	0.2	29.99	1.92E-05
(GO:0019843)	rRNA binding	8	0.3	26.24	6.02E-07
(GO:0070717)	poly-purine tract binding	3	0.12	24.23	3.13E-02
(GO:0003730)	mRNA 3'-UTR binding	10	0.45	22.34	3.08E-08
(GO:0000049)	tRNA binding	7	0.31	22.27	1.36E-05
(GO:0003724)	RNA helicase activity	8	0.38	21.26	2.59E-06
(GO:0017069)	snRNA binding	4	0.2	20	7.85E-03
(GO:0003725)	double-stranded RNA binding	6	0.35	17.02	3.15E-04
(GO:0003729)	mRNA binding	19	1.31	14.51	9.90E-14
(GO:0004386)	helicase activity	11	0.79	13.91	2.86E-07
(GO:0003723)	RNA binding	83	8.03	10.34	5.76E-69

*GO, Gene ontology; molecular function complete, http://geneontology.org/

** FDR corrected p-value (Kolmogorov-Smirnov test)

Supplementary Notes

Supplementary Note 1: Clinical case reports

We reported previously the complete clinical details on K8100 [1]. A summary of the clinical findings at last clinical examination for two affected males in K8100 (IV-1 and IV-2) and four unrelated affected males in K9648, K9656, K9667, K9677 are summarized in Table 1.

Family K8100. Affected males IV-1 and IV-2, ages 28 and 24 years, respectively, have shown lifelong cognitive impairment requiring special education throughout their school years. They have short stature and head size >80th centile. The elder brother has prominence of the forehead, downslanting palpebral fissures, short philtrum, a vaulted palate, placement of two mandibular incisors behind the other two, large jaw, small hands and small feet. He has generalized stiffness at all joints, limited extension of all large joints and asymmetric reflexes. Grand mal seizures occurred from years 3 to 8 years. He has myopia, repaired cleft palate and unilateral cryptorchidism. He has an even-tempered demeanor, is friendly and sedentary. The younger brother has a similar developmental history and growth pattern except that he has a lower body mass index (BMI) than his brother. His forehead was not prominent, palpebral fissures were minimally downslanted, alveolar ridges were thick, palate narrow and vaulted, and his teeth were crowded. He has myopia, low posterior hairline, webbed neck, small hands and feet, lower limb hyperreflexia, joint limitation, petit mal seizures and behavioral problems with aggressiveness.

Family K9648. The proband is the only affected male in this kindred. At birth, he had normal length and weight measurements, Tetralogy of Fallot, horseshoe kidney and cryptorchidism. He has prominent forehead, bitemporal narrowing, proptosis, hypotelorism, Axenfeld-Rieger anomaly with glaucoma, large prominent left ear, tubular nose and prominent lips, single central incisor, hypodontia, coxa valga, kyphoscoliosis, small hands and feet, a sacral dimple and a lipoma over the lower spine. His development was globally delayed, his height fell below the third centile, he required G-tube feedings, he had no speech and uses a wheelchair in mid-childhood.

Family K9656. The affected male in this kindred had birth measurements in the normal range, a small atrial septal defect, unilateral renal agenesis and a two vessel cord. He has hemangiomas between the brows and at the back of the neck, epicanthus, infraorbital creases, wide nasal root and a short nose. The ears are slightly posteriorly rotated. He has hypotonia and increased joint

flexibility as well as fleshy hands and fingers. He has micropenis and a small scrotum, and a history of inguinal hernia. Development has been globally delayed. He has a history of failure to thrive, up to age 5 ½ years, with height and weight below the 3rd centiles but a preserved head circumference. By age 10 years he developed obesity and gynecomastia. Despite significantly delayed milestones, his intellectual disability is mild.

Family K9667. The affected male had birth measurements in the 20th-25th centiles and cryptorchidism at birth. He had normal growth, small hands and feet, exotropia, myopia, excessively folded helices, a bulbous nose, hiatal hernia and a sacral dimple with tethered cord. He had hypotonia and hypertonia, drooling, dysphagia, constipation, and jerky limb movements that do not appear to be seizures. He had global developmental delay and at 8 years had an IQ below 50, no speech, walked with a gait trainer, and experienced sleep disturbance, and incontinence.

Family K9677. The affected male in this kindred was large at birth and had imperforate anus. He had macrocephaly, facial hemangiomas, exotropia, keratoconus, nystagmus, excessively folded helices, wide nasal tip, hypodontia, micrognathia, umbilical hernia, and bilateral clubfoot. His development was globally delayed, he developed seizures at age 9 years, had hypotonia, tremor of the hands and small hands and feet. He had mild intellectual disability, lived in a group home, had useful speech, independent ambulation and experiences mood lability and impulsiveness.

Supplementary References

1 Armfield, K. *et al.* X-linked mental retardation syndrome with short stature, small hands andfeet, seizures, cleft palate, and glaucoma is linked to Xq28. *Am J Med Genet* **85**, 236-242 (1999).
