# Supplementary material for: Mutations in FAM50A suggest that Armfield XLID syndrome is a spliceosomopathy
Source: Nat Commun. 2020 Jul 23;11:3698. doi: 10.1038/s41467-020-17452-6 (PMC7378245; doi:10.1038/s41467-020-17452-6)
Supplement: Supplementary file 3 — Description of Additional Supplementary Files [file 41467_2020_17452_MOESM3_ESM.pdf]

Description of Additional Supplementary Files

File name: Supplementary Data 1

Description: List of proteins identified by FAM50A pulldown and mass spectrometry analysis.
