# Supplementary material for: Mutations in FAM50A suggest that Armfield XLID syndrome is a spliceosomopathy
Source: Nat Commun. 2020 Jul 23;11:3698. doi: 10.1038/s41467-020-17452-6 (PMC7378245; doi:10.1038/s41467-020-17452-6)
Supplement: Supplementary file 5 — Reporting Summary [file 41467_2020_17452_MOESM5_ESM.pdf]

nature research

Corresponding author(s): Charles E. Schwartz, Erica E. Davis, Cheol-Hee Kim

Last updated by author(s): May 21, 2020

Reporting Summary

Nature Research wishes to improve the reproducibility of the work that we publish. This form provides structure for consistency and transparency in reporting. For further information on Nature Research policies, seeAuthors & Referees and theEditorial Policy Checklist .

Statistics

For all statistical analyses, confirm that the following items are present in the figure legend, table legend, main text, or Methods section.

n/a	Confirmed	
☐	☒	The exact sample size (*n*) for each experimental group/condition, given as a discrete number and unit of measurement
☐	☒	A statement on whether measurements were taken from distinct samples or whether the same sample was measured repeatedly
☐	☒	The statistical test(s) used AND whether they are one- or two-sided<br>*Only common tests should be described solely by name; describe more complex techniques in the Methods section.*
☒	☐	A description of all covariates tested
☐	☒	A description of any assumptions or corrections, such as tests of normality and adjustment for multiple comparisons
☐	☒	A full description of the statistical parameters including central tendency (e.g. means) or other basic estimates (e.g. regression coefficient) AND variation (e.g. standard deviation) or associated estimates of uncertainty (e.g. confidence intervals)
☐	☒	For null hypothesis testing, the test statistic (e.g. *F*, *t*, *r*) with confidence intervals, effect sizes, degrees of freedom and *P* value noted<br>*Give P values as exact values whenever suitable.*
☒	☐	For Bayesian analysis, information on the choice of priors and Markov chain Monte Carlo settings
☒	☐	For hierarchical and complex designs, identification of the appropriate level for tests and full reporting of outcomes
☒	☐	Estimates of effect sizes (e.g. Cohen's *d*, Pearson's *r*), indicating how they were calculated

Our web collection on statistics for biologists contains articles on many of the points above.

Software and code

Policy information about availability of computer code

Data collection	Nikon imaging software (NIS-element AR 64-bit version 3.21; Laboratory Imaging), Vertebrate Automated Screening Technology (VAST; Union Biometrica) Bioimager platform, (ZEN Pro 2.3 software; Zeiss)
Data analysis	CASAVA 1.8.2 (Illumina, San Diego, CA), BWA 0.6.2-r126, samtools 0.1.18, Picard 1.79, UnifiedGenotyper, VariantRecalibrator, GATK 2.2, GeneScan software (Applied Biosystems), I-TASSER (https://zhanglab.ccmb.med.umich.edu/I-TASSER/), YASPIN, mCSM (http://biosig.unimelb.edu.au/mcsm/), SDM (http://marid.bioc.cam.ac.uk/sdm2/), DUET (http://biosig.unimelb.edu.au/duet/), SAAFEC (http://compbio.clemson.edu/SAAFEC/), FOLDX (http://foldxsuite.crg.eu/), cNLS mapper: (http://nls-mapper.iab.keio.ac.jp/cgi-bin/NLS_Mapper_form.cgi), cNLStradamus: (http://www.moseslab.csb.utoronto.ca/NLStradamus), Image Studio Lite v5.2,Optimized CRISPR Design (http://crispr.mit.edu/), DNASTAR (Lasergene 17 software), Image J(NIH) v1.52, GraphPad Prism 8, Microsoft Excel 16.36, Tape Station Analysis Software (Agilent), Bcl2Fastq v2.20 conversion software (Illumina), TrimGalore toolkit v0.6.5 (http://www.bioinformatics.babraham.ac.uk/projects/trim_galore), Cutadapt 2.10, STAR RNA-seq alignment tool v2.7.0, HTSeq tool (https://htseq.readthedocs.io/en/master/), DESeq2 , Bioconductor package with the R statistical programming environment (https://www.r-project.org/), rMATS algorithm 3.0.8, DAVID pathway analysis v6.8 (https://david.ncifcrf.gov/), online nanoLC-MS/MS, Proteome Discoverer v. 1.4 (Thermo), Mascot v. 2.2 (Matrix Science), Percolator 3.0, SAINTexpress

For manuscripts utilizing custom algorithms or software that are central to the research but not yet described in published literature, software must be made available to editors/reviewers. We strongly encourage code deposition in a community repository (e.g. GitHub). See the Nature Research guidelines for submitting code & software for further information.

Data

Policy information about availability of data

All manuscripts must include a data availability statement. This statement should provide the following information, where applicable:

- Accession codes, unique identifiers, or web links for publicly available datasets
- A list of figures that have associated raw data
- A description of any restrictions on data availability

GenBank: (https://www.ncbi.nlm.nih.gov), gnomAD: (https://gnomad.broadinstitute.org/), zebrafish baseline RNA-seq data: (https://www.ebi.ac.uk/gxa/experiments/E-ERAD-475/Results), Human Phenotype Ontology: (HPO, https://hpo.jax.org/), Online Mendelian Inheritance in Man: (OMIM, https://omim.org/), gene ontology: (GO, http://geneontology.org/), Zebrafish Center for Disease Modeling: (https://cc.aris.re.kr/zcdm), Ensembl: (http://ensembl.org/),ClinVar database (https://www.ncbi.nlm.nih.gov/clinvar/), iP Tree: http://203.64.84.190:8080/IPTREEr/iptree.htm,SIFT: https://sift.bii.a-star.edu.sg/, Mutation Taster: http://www.mutationtaster.org/, I-Mutant: http://gpcr2.biocomp.unibo.it/cgi/predictors/I-Mutant3.0/I-Mutant3.0.cgi,PolyPhen: http:genetics.bwh.harvard.edu/pph2/, Consurf: http://consurf.tau.ac.il/2016/, CADD: https://cadd.gs.washington.edu/, KEGG pathway: (https://www.genome.jp/kegg/pathway.html)

Data availability statement: Consent restrictions preclude deposition of X-chromosome sequencing data (family K8100) or whole exome sequencing data (families K9648, K9656, K9667, or K9677). However, specific information (e.g., specific variants, but not full datasets) can be obtained upon request from the corresponding authors. All FAM50A variants have been deposited in the ClinVar database under accession numbers VCV000872936.1 [https://www.ncbi.nlm.nih.gov/clinvar/variation/872936/], VCV000872937.1 [https://www.ncbi.nlm.nih.gov/clinvar/variation/872937/], VCV000872938.1 [https://www.ncbi.nlm.nih.gov/clinvar/variation/872938/], VCV000872939.1 [https://www.ncbi.nlm.nih.gov/clinvar/variation/872939/] and VCV000872940.1 [https://www.ncbi.nlm.nih.gov/clinvar/variation/872940/]. Transcriptomic data were deposited in the NCBI Gene Expression Omnibus under accession numbers GEO: GSE145711 (zebrafish [https://www.ncbi.nlm.nih.gov/geo/query/acc.cgi?acc=GSE145711]) and GSE145710 (human [https://www.ncbi.nlm.nih.gov/geo/query/acc.cgi?acc=GSE145710). Proteomic data were deposited in the ProteomeXchange database under accession number PXD017642 [http://www.ebi.ac.uk/pride/archive/projects/PXD017642]. The source data underlying Figs. 3b, c, 6c, d and Supplementary Figs. 3a, b, 5a, 6d, f, 10a, b, c, d, e, 11b, d, 12, 13b, and 17b, d are provided as a Source data file.

Field-specific reporting

Please select the one below that is the best fit for your research. If you are not sure, read the appropriate sections before making your selection.

- [x] Life sciences
- [ ] Behavioural & social sciences
- [ ] Ecological, evolutionary & environmental sciences

For a reference copy of the document with all sections, see nature.com/documents/nr-reporting-summary-flat.pdf

Life sciences study design

All studies must disclose on these points even when the disclosure is negative.

Sample size	Sample size was based on previous studies with similar scope, which informed expected effect size and variability within the sample. Feasibility of experiments involving animals also informed the selection of sample size.
Data exclusions	No data were excluded from the analysis in the current study.
Replication	Genetic studies: discovery by candidate sequencing or WES with independent confirmation; RT-PCR: 2x; FAM50A localization in NIH/3T3 cells: 1x, 31 cells evaluated; variant localization in COS-7: 3x; WISH: at least 9x, and up to 182 embryos were evaluated; in vivo complementation: at least 2x; zebrafish transcriptomes: 5x; LCL transcriptomes: 3x; proteomics: 2x.
Randomization	Randomization was used when possible: running nextgen libraries on random lanes to avoid batch effects.
Blinding	Where possible, the investigator was blind to experimental condition at the time of treatment and data collection (e.g. cell transfection, embryo microinjection, larval imaging, larval measurement), for statistical analysis, unblinding was required to perform meaningful comparisons.

Reporting for specific materials, systems and methods

We require information from authors about some types of materials, experimental systems and methods used in many studies. Here, indicate whether each material, system or method listed is relevant to your study. If you are not sure if a list item applies to your research, read the appropriate section before selecting a response.

Materials & experimental systems

n/a	Involved in the study	
☐	☒	Antibodies
☐	☒	Eukaryotic cell lines
☒	☐	Palaeontology
☐	☒	Animals and other organisms
☐	☒	Human research participants
☐	☒	Clinical data

Methods

n/a	Involved in the study	
☒	☐	ChIP-seq
☒	☐	Flow cytometry
☐	☒	MRI-based neuroimaging

Antibodies

Antibodies used	Localization of endogenous FAM50A protein in NIH/3T3 cells: anti-human FAM50A antibody (1:200; Novus Biologicals, NBP1-89344); Alexa Fluor 488-conjugated goat anti-rabbit secondary antibody (1:1,000; Invitrogen, A-11008) and Hoechst 33342 (1:10,000; Invitrogen, H3570); FAM50A immunoblotting in human LCLs and zebrafish: rabbit anti-FAM50A antibody (1:900, Novus Biologicals; NBP1-89344) and mouse anti-GAPDH antibody (1:3,000, Santa Cruz Biotechnology, sc-47724); anti-rabbit IgG-HRP (1:4,000, Santa Cruz Biotechnology, sc-2357) and anti-mouse IgGκ-HRP (1:4,000, Santa Cruz Biotechnology, sc-516102) Localization of tagged WT and mutant FAM50A in COS-7 cells: anti-V5 mouse monoclonal antibody (1:500, Invitrogen, R960-25); Alexa Fluor 594 anti-Mouse IgG (1:1,000, Invitrogen, A32742) and Alexa Fluor 488 Phalloidin (1:40, Invitrogen, A12379); Immunostaining on whole-mount zebrafish larvae: FAM50A antibody (1:200; Novus Biologicals, NBP1-89344); Alexa Fluor 568-conjugated secondary antibodies (1:500; Life Technologies). For nuclei staining,Hoechst 33342 at 1 mg/ml and 10 µg/ml; Mitotic cell cycle progression studies in whole-mount larvae; anti-phospho-histone 3 (PH3; 1:500, Santa Cruz Biotechnology, sc-8656-R); 594 anti-rabbit IgG, ThermoFisher; 1:500 Co-immunoprecipitation (co-IP) using in vitro cell models; IP: anti-FAM50A antibody (1:111, Novus Biologicals; NBP1-89344); Transfection and sample integrity: (anti-GAPDH antibody, 1:4,000, Santa Cruz Biotechnology, sc-47724) and (anti-V5 antibody, 1:3,000, ThermoFisher Scientific; R960-25).
Validation	FAM50A antibody: Validated on fam50a KO zebrafish (see Supplementary Fig 5); GAPDH antibody: Cited in 834 publications for WB according to the product web page; V5 antibody: according to the product web page, used in IF in 99 publications and used in coIP in 116 publications; phospho-Histone H3: according to the product web page, cited in 70 publications.

Eukaryotic cell lines

Policy information about cell lines

Cell line source(s)	NIH3T3 cells, lymphoblast cell lines, Cos-7 cells, U-87 glioblastoma cells, HEK293T cells (American Tissue Culture Collection); Lymphocyte cell lines (derived from males with FAM50A mutations and healthy control)
Authentication	Commercially available cell lines were not authenticated; LCLs were authenticated by genotyping the FAM50A mutation.
Mycoplasma contamination	The cell line used in current study were not tested for mycoplasma contamination.
Commonly misidentified lines (See ICLAC register)	We did not use any commonly misidentified cell lines.

Animals and other organisms

Policy information about studies involving animals; ARRIVE guidelines recommended for reporting animal research

Laboratory animals	We utilized zebrafish (Danio rerio) as an animal model. We used both male and female zebrafish on wild-type or transgenic (tg,huc:egfp; tg,kdrl:egfp; tg,col1a1:egfp) background. We used 3-12 month old zebrafish adults for natural mating to generate embryos for experiments. For phenotyping studies, we used embryos aged from 1-cell stage to 5 day post fertilization larvae.
Wild animals	The study did not involve any wild animals.
Field-collected samples	The study did not involve samples collected from the field.
Ethics oversight	Animal experiments were conducted according to approved guidelines and regulations of the Institutional Animal Care and Use Committee (IACUC Protocol A154-18-06 at Duke University and IACUC protocol IS00013481 at Northwestern University) or the Animal Ethics Committee of Chungnam National University (CNU-00866).

Note that full information on the approval of the study protocol must also be provided in the manuscript.

Human research participants

Policy information about studies involving human research participants

Population characteristics	The study involves five unrelated families of different ethnic backgrounds (four families, N. European origin; one family, Mixed (African-American, Middle Eastern, Mixed European). In total we identified nine male individuals of different age groups, who were diagnosed with clinical features of Armfield XLID syndrome. FAM50A genotype and participant age are as follows: Individual K8100 IV-1, c.764A>G; p.Asp255Gly (28 years) ; K8100 IV-2 c.764A>G; p.Asp255Gly (24 years); K9648, c.616T>G; p.Trp206Gly (10 years); K9656, c.761A>G; p.Glu254Gly (10 years), K9667, c.817C>T; p.Arg273Trp (8 years); K9677, c.763G>A; p.Asp255Asn (9 years).
Recruitment	The current study recruited human cases from unrelated families showing clinical symptoms of Armfield XLID syndrome. All participants in this study were assessed clinically by local physicians with medical genetics expertise. The only bias was that all participant families agreed to participate in research; this is unlikely to affect results since we do not report incidence of this disorder in the population.
Ethics oversight	Targeted sequencing and whole exome sequencing were approved by the relevant institutional ethics committees at each participating center (Greenwood Genetic Center; A.I. duPont Hospital for Children; McMaster University Medical Center; Phoenix Children's Medical Group; University of North Carolina School of Medicine). We obtained signed informed consent for study procedures, publication of genetic findings, and photographs from all participants or their legal representatives.

Note that full information on the approval of the study protocol must also be provided in the manuscript.

Clinical data

Policy information about clinical studies

All manuscripts should comply with the ICMJEguidelines for publication of clinical research and a completedCONSORT checklist must be included with all submissions.

Clinical trial registration	Not applicable
Study protocol	Not applicable
Data collection	Not applicable
Outcomes	Not applicable

Magnetic resonance imaging

Experimental design

Design type	Not applicable
Design specifications	Not applicable
Behavioral performance measures	Not applicable

Acquisition

Imaging type(s)	Not applicable
Field strength	Not applicable
Sequence & imaging parameters	Not applicable
Area of acquisition	Not applicable

Diffusion MRI ☐ Used ☐ Not used

Preprocessing

Preprocessing software	Not applicable
Normalization	Not applicable
Normalization template	Not applicable
Noise and artifact removal	Not applicable
Volume censoring	*Define your software and/or method and criteria for volume censoring, and state the extent of such censoring.*

Statistical modeling & inference

Model type and settings	Not applicable
Effect(s) tested	Not applicable

Specify type of analysis: ☐ Whole brain ☐ ROI-based ☐ Both

Statistic type for inference (See Eklund et al. 2016)	Not applicable
Correction	Not applicable

Models & analysis

n/a	Involved in the study
☒	☐ Functional and/or effective connectivity
☒	☐ Graph analysis
☒	☐ Multivariate modeling or predictive analysis
